# Genome-wide screening for DNA variants associated with reading and language traits

**DOI:** 10.1111/gbb.12158

**Published:** 2014-08-29

**Authors:** A Gialluisi, D F Newbury, E G Wilcutt, R K Olson, J C DeFries, W M Brandler, B F Pennington, S D Smith, T S Scerri, N H Simpson, The SLI Consortium, M Luciano, D M Evans, T C Bates, J F Stein, J B Talcott, A P Monaco, S Paracchini, C Francks, S E Fisher

**Affiliations:** † Language and Genetics Department, Max Planck Institute for PsycholinguisticsNijmegen, The Netherlands; ‡ Wellcome Trust Centre for Human Genetics, University of OxfordOxford, UK; § Institute for Behavioral Genetics, University of ColoradoBoulder, CO, USA; ¶ Department of Psychology and Neuroscience, University of ColoradoBoulder, CO, USA; ** MRC Functional Genomics Unit, University of OxfordOxford, UK; †† Department of Psychology, University of DenverDenver, CO, USA; ‡‡ Munroe Meyer Institute, University of Nebraska Medical CenterOmaha, NE, USA; §§ The Walter and Eliza Hall Institute of Medical ResearchMelbourne, VIC, Australia; ¶¶ The members of the SLIC are listed in the Acknowledgments section; *** Department of Psychology, Centre for Cognitive Aging and Cognitive Epidemiology, University of EdinburghEdinburgh, UK; ††† MRC Integrative Epidemiology Unit, University of BristolBristol, UK; ‡‡‡ School of Social and Community Medicine, University of BristolBristol, UK; §§§ University of Queensland Diamantina Institute, Translational Research InstituteBrisbane, QLD, Australia; ¶¶¶ Genetic Epidemiology, Queensland Institute of Medical ResearchBrisbane, QLD, Australia; **** Department of Physiology, University of OxfordOxford, UK; †††† School of Life and Health Sciences, Aston UniversityBirmingham, UK; ‡‡‡‡ School of Medicine, University of St AndrewsSt Andrews, UK; §§§§ Tufts UniversityMedford, MA, USA; ¶¶¶¶ Donders Institute for Brain, Cognition & BehaviorNijmegen, The Netherlands

**Keywords:** pleiotropic variants, CLDRC, developmental dyslexia, GWAS, language, meta-analysis, reading, reading disability, SLIC, specific language impairment

## Abstract

Reading and language abilities are heritable traits that are likely to share some genetic influences with each other. To identify pleiotropic genetic variants affecting these traits, we first performed a genome-wide association scan (GWAS) meta-analysis using three richly characterized datasets comprising individuals with histories of reading or language problems, and their siblings. GWAS was performed in a total of 1862 participants using the first principal component computed from several quantitative measures of reading- and language-related abilities, both before and after adjustment for performance IQ. We identified novel suggestive associations at the SNPs rs59197085 and rs5995177 (uncorrected *P* ≈ 10^–7^ for each SNP), located respectively at the *CCDC136*/*FLNC* and *RBFOX2* genes. Each of these SNPs then showed evidence for effects across multiple reading and language traits in univariate association testing against the individual traits. *FLNC* encodes a structural protein involved in cytoskeleton remodelling, while *RBFOX2* is an important regulator of alternative splicing in neurons. The *CCDC136/FLNC* locus showed association with a comparable reading/language measure in an independent sample of 6434 participants from the general population, although involving distinct alleles of the associated SNP. Our datasets will form an important part of on-going international efforts to identify genes contributing to reading and language skills.

Reading disability (RD, also known as developmental dyslexia) refers to a significant difficulty in reading that cannot be explained by obvious causes, such as sensory impairments or lack of educational opportunity (Shaywitz *et al.* 1990). Specific language impairment (SLI) is diagnosed as an unexpected difficulty or delay in acquiring spoken language abilities, despite normal hearing and intelligence, and in absence of overt neurological deficits (Bishop 1994). RD and SLI are among the most prevalent neurocognitive disorders of school-aged children, with prevalence ≈5–8% in many populations (Shaywitz *et al.* 1990; Tomblin *et al.* 1997). Both are complex disorders with moderate to high heritabilities (30–70%) as assessed by studies of families and twins (Barry *et al.* 2007; Fisher & DeFries 2002).

RD and SLI display high comorbidity: 43% of SLI children are later diagnosed with RD and up to 55% of dyslexic children meet criteria for SLI (McArthur *et al.* 2000; Snowling *et al.* 2000). Moreover, RD and SLI show comorbidity with other neurodevelopmental traits including attention deficit hyperactivity disorder (ADHD) (Pennington 2006; Willcutt *et al.* 2010) and speech sound disorders (Newbury & Monaco 2010; Pennington & Bishop 2009). It is likely that these disorders arise due to some shared genetic/neurobiological mechanisms, as well as non-shared causal factors (Newbury *et al.* 2011; Paracchini 2011). A study of twins by Harlaar *et al.* (2008) indicated that an association between early language and later reading is underpinned by common environmental and genetic influences, and a family study by Logan *et al.* (2011) also found significant genetic correlations of reading and language measures.

Variants of several genes have previously been associated with RD, most notably *DYX1C1* (15q21, Taipale *et al.* 2003), *KIAA0319* and *DCDC2* (6p22, Cope *et al.* 2005; Francks *et al.* 2004; Meng *et al.* 2005), *MRPL19/C2ORF3* (2p12, Anthoni *et al.* 2007) and *ROBO1* (3p12, Bates *et al.* 2011; Hannula-Jouppi *et al.* 2005). Similarly, some loci have been implicated in SLI; variants in genes, such as *CNTNAP2* (7q36, Vernes *et al.* 2008) and *CMIP* and *ATP2C2* (16q23-24, Newbury *et al.* 2009) show associations with quantitative traits in children with typical SLI, while rare mutations of *FOXP2* (7q31, Fisher & Scharff 2009) cause a monogenic speech and language disorder. These genes were mostly identified through linkage analysis followed by either positional cloning or else targeted association mapping. Functional analyses suggest that some of these genes mediate important processes in central nervous system (CNS) development, such as neuronal migration, axonal guidance and neurite outgrowth (Carrion-Castillo *et al.* 2013; Vernes *et al.* 2011). A subset of the candidate genes may contribute to both RD and SLI, again indicating a partial genetic overlap for these traits (Bates *et al.* 2011; Newbury *et al.* 2011; Scerri *et al.* 2011b). Crucially, an overwhelming majority of the heritable variance in reading and language skills is unexplained, and the molecular mechanisms that contribute to RD and SLI remain largely unknown (Newbury & Monaco 2010; Peterson & Pennington 2012).

Some of the genetic variation contributing to RD and SLI is also likely to impact on reading/language skills in the general population (Bates *et al.* 2011; Luciano *et al.* 2007; Paracchini *et al.* 2008, 2011; Scerri *et al.* 2011b; Whitehouse *et al.* 2011). To detect previously undiscovered associations of common genetic variants with reading and language skills, it is therefore appropriate to sample broad ranges of the trait distributions in study datasets, while screening over the entire genome.

In recent years, a small number of studies have tried to identify genes involved in reading and/or language through genome-wide association scanning (GWAS). An early GWAS for reading ability used DNA pooling of low vs. high reading ability groups in ∼1500 7-year-old children, and a relatively low density SNP microarray with ∼107,000 SNPs (Meaburn *et al.* 2008). The SNPs showing the largest allele frequency differences between low and high ability groups were further genotyped and tested in an additional sample of 4,258 children, with 10 SNPs finally showing nominally significant association with continuous variation in reading ability (Meaburn *et al.* 2008). A GWAS on mismatch negativity, which is a potential endophenotype of dyslexia derived from electroencephalography, has also been reported based on 386 dyslexic children, and showed replicable association of the SNP rs4234898 on 4q32 along with the haplotype rs4234898-rs11100040 (Roeske *et al.* 2011). These were shown to affect mRNA expression levels of *SLC2A3* (12p13), which codes for a neuronal glucose transporter, suggesting a possible role of glucose levels in memory performance necessary for speech perception in dyslexia (Roeske *et al.* 2011). More recently, a genome-wide linkage and association scan using ∼133,000 SNPs, in 718 subjects from 101 dyslexia-affected families, reported a borderline significant association with dyslexia status at rs9313548, near *FGF18* (5q35.1), which is a gene involved in laminar positioning of cortical neurons during development (Field *et al.* 2013).

Two GWAS studies have directly attempted to identify shared genetic contributions to reading and language abilities. Luciano *et al.* (2013), in a GWAS on quantitative reading and language traits in two population datasets (*N* ∼ 6500), found the strongest association between rs2192161, in the *ABCC13* pseudogene (21q11.2), and a nonword repetition measure (p ∼ 7 × 10^–8^), while rs4807927 (*DAZAP1*, 19p13.3) showed association with both word reading and a composite reading–spelling factor score (p ∼ 10^–6^ for both traits). In the same study, *CDC2L1*, *CDC2L2*, *LOC728661* (1p36.33) and *RCAN3* (1p36.11) showed significant gene-based associations with the reading–spelling factor (Luciano *et al.* 2013). A case-control GWAS using a relatively small number of RD (*N* = 353), language impairment (*N* = 163) and comorbid cases (*N* = 174), in comparison to general population controls (*N* = 4117), identified nominally significant associations for the comorbid cases at rs12636438 and rs1679255 in *ZNF385D* (3p24.3) (Eicher *et al.* 2013). These SNPs also showed associations with a vocabulary measure and white matter volumes of brain fibre tracts previously implicated in language, in an independent dataset (Eicher *et al.* 2013).

In the present study, we carried out a GWAS meta-analysis for genetic variants influencing reading and language abilities. We included three long-established datasets comprising children with reading or language problems, along with their siblings. This approach complemented other recent GWAS studies of reading/language performance (Eicher *et al.* 2013; Luciano *et al.* 2013) because it included continuous trait variance across a broad range of reading and language abilities, but also involved a pronounced enrichment for poor performance while not applying an arbitrary dichotomy between RD/SLI cases and controls.

Within each dataset, we tested single nucleotide polymorphisms (SNPs), along with single base insertions/deletions (indels), for association with the first Principal Component (PC) derived from a range of reading- and language-related quantitative traits. We then meta-analysed the GWAS results from the separate datasets, followed by gene- and pathway-level analysis, and we checked the most significant associations arising from our analysis within the GWAS results generated by Luciano *et al.* (2013).

Although we used PC-based analysis as a form of data reduction for the purposes of GWAS, we also investigated the two most significant SNP associations arising from our meta-analysis by using multivariate association modelling in each dataset, and by testing of these SNPs against the individual measures separately. This approach would help to understand the cross-phenotypic effects involved. In other words, the PC-based GWAS was used to identify potential genetic effects on shared variance between multiple reading and language measures, and then pleiotropy was investigated in more detail through univariate analysis and multivariate modelling, for individual SNPs implicated by the PC-based GWAS meta-analysis. In addition, in order to more closely match the trait measurement across all datasets, we repeated the GWAS and meta-analysis using the first PC of only single word reading and spelling ability, because these were the only two measures available in all datasets.

Some genetic effects on reading and language may be pleiotropic for IQ, whereas other effects may be largely or wholly independent of IQ (Bishop & Snowling 2004; Pennington & Bishop 2009). To detect the latter type of effect it is advantageous to remove the shared variance with IQ that is present in measures of reading and language, prior to association testing. We therefore performed our GWAS analyses both with and without IQ-adjustment of the reading and language measures. In addition, Luciano *et al.* (2013) analysed only IQ-adjusted data, so that for cross-comparing of results an IQ-adjustment was desirable to include in this study.

## Subjects and methods

### Datasets

#### UK Reading Disability (UK-RD)

This dataset comprised children diagnosed with RD, and their siblings, collected at the Dyslexia Research Centre clinics in Oxford and Reading, or the Aston Dyslexia and Development Clinic in Birmingham, UK. Ethical approval was acquired from the Oxfordshire Psychiatric Research Ethics Committee (OPREC O01.02) and written informed consent of the participants (or their parents) was obtained. The total number of participants was 983, mean age 11.7 years, age range 5–31, from 608 independent nuclear families. All children, regardless of diagnosis, were administered psychometric tests of reading- and language-related abilities, as well as assessments of verbal and non-verbal IQ (details further below). A subset of this dataset has been analysed in previous studies on reading (Becker *et al.* 2013) and handedness traits (Brandler *et al.* 2013; Scerri *et al.* 2011a), but no GWAS of reading-/language-related traits has previously been reported.

#### SLI Consortium (SLIC)

The SLI Consortium dataset comprised children affected by SLI, along with their siblings, recruited from five centres across the UK; The Newcomen Centre at Guy's Hospital, London (now called Evelina Children's Hospital); the Cambridge Language and Speech Project (CLASP); the Child Life and Health Department at the University of Edinburgh; the Department of Child Health at the University of Aberdeen and the Manchester Language Study, as described in previous reports by the SLI Consortium (Falcaro *et al.* 2008; Newbury *et al.* 2009; The SLI Consortium 2002, 2004). This sample included 49 families from the Guy's Hospital, London cohort which had not been included in previous SLI Consortium studies. Ethical agreement was given by local ethics committees of the hospitals involved in the consortium, and all subjects provided informed consent. All children in this sample were assessed for a number of reading- and language-related traits (see below) regardless of their language ability. For this study, we obtained genome-wide genotype data for affected probands and their available siblings, for a total of 548 participants, mean age 10 years, age range 5–19, from 288 independent nuclear families. The SLIC dataset has been used for prior linkage studies (Falcaro *et al.* 2008; The SLI Consortium 2002, 2004), and targeted candidate gene analyses (Newbury *et al.* 2009; Vernes *et al.* 2008). More recently, it has been used for investigating copy number variants (Ceroni *et al.* 2014), identification of chromosomal abnormalities (Simpson *et al.* 2014) and in a genome-wide search for parent-of-origin effects on SLI (Nudel *et al.* 2014). However, no GWAS for continuous language and reading scores has yet been reported for this (or any other) SLI sample.

#### Colorado Learning Disabilities Research Centre (CLDRC)

The Colorado Learning Disabilities Research Centre (CLDRC) dataset was derived from an ongoing study on the aetiology of learning disabilities run in 27 school districts in Colorado, USA (DeFries *et al.* 1997; Willcutt *et al.* 2005). Pairs of twins were initially recruited based on a school report of RD, ADHD or other learning disabilities in one or both of the twins; they were then administered a number of psychometric tests for several learning-related skills, along with their additional co-siblings, and DNA was collected for genetic studies. The Institutional Review Boards of the University of Nebraska Medical Center and of the University of Colorado at Boulder had approved the protocol, and written informed consent of the participants (or their parents) was obtained.

For this study, for MZ twin pairs, we selected one child per pair based on the maximum availability of reading- and language-related trait data, or otherwise randomly. The sample of twins and siblings available for this study comprised 749 participants in total, mean age 11.7 years, age range 8–19, from 343 unrelated twinships/sibships. Of these, 266 of the twinships/sibships (a total of 585 participants) were originally recruited via a proband with a history of RD, and 77 of the twinships/sibships (164 participants in total) were originally recruited via a proband with a history of ADHD. We analysed these two subsets separately for GWAS before meta-analysing the results together with those from the other datasets listed above. The two subsets are indicated hereafter as CLDRC-RD and CLDRC-ADHD. As for the other datasets, no prior GWAS has been reported.

### Genotype data generation, quality control (QC) and imputation

DNA was extracted from whole blood or buccal swab samples and prepared for genotyping using standard protocols. Genome-wide genotype data were generated for each dataset using Illumina® SNP arrays. These were the HumanHap 550k for a first genotyping wave of 200 subjects from UK-RD, and the Human OmniExpress (730k SNPs) for SLIC, CLDRC and the remaining UK-RD samples. Data were processed using Illumina's BeadStudio®/GenomeStudio® software, following the manufacturer's guidelines. All datasets then underwent a first round of quality control, using functions in the software PLINK v1.07 (Purcell *et al.* 2007; http://pngu.mgh.harvard.edu/∼purcell/plink/), in which all SNPs deviating from Hardy–Weinberg Equilibrium (HWE, *P* < 1 × 10^–6^), with Minor Allele Frequency (MAF) < 1% and call frequency < 99%, were filtered out. In addition, samples were excluded if they showed inconsistencies in genome-wide identity-by-descent sharing with their siblings and unrelated individuals, or sex mismatches, or call rates <98%. Multi-dimensional scaling (MDS) analysis of genome-wide genotype data was used to identify any subjects that did not cluster together with the majority of the dataset, and these were discarded, as were any outliers for genome-wide homozygosity. These QC steps were followed by genotype phasing using MACH v1.0 (Liu *et al.* 2010; http://www.sph.umich.edu/csg/abecasis/MACH/index.html) and imputation of SNPs and single-base indels using Minimac (Howie *et al.* 2012; http://genome.sph.umich.edu/wiki/Minimac), with the 1000 Genomes Project reference dataset (GIANT all populations panel, Phase 1, v3; The 1000 Genomes Project Consortium, 2012; http://www.1000genomes.org). We excluded poorly imputed polymorphisms (with r^2^ < 0.3), and deleted individual genotypes with imputation quality scores <0.9. A final quality control procedure was then run on the imputed data, using PLINK, in which we discarded SNPs with HWE *P* < 5 × 10^–6^, MAF < 1%, and call frequency <95%. Key features of the QC are shown in Table1. Further details are reported in Appendix S4.

**Table 1:** Genotype quality control (QC) filters used and number of samples/markers discarded at each step (see Methods and Appendix S4 for details)

QC step	CLDRC (749)†	UK-RD (200 + 818)‡	SLIC (548)
HWE *P* < 1 × 10^–6^ (SNPs)	57	12,631§; 191	54
MAF < 1% (SNPs)	74,770	23,467; 77,342	1,718
Call freq < 99% (SNPs)	0 ¶	82,052; 0¶	72,043
Call rate < 98% (samples)	0 ¶	3; 0 ¶	9
IBD sharing (samples)	11	1; 7	17
Sex mismatch (samples)	3	0; 8**	13††
Homozygosity outlier (samples)	6	1; 3	2
MDS outlier (samples)	0	0; 2	5
HWE *P* < 5 × 10^–6^ (SNPs)*	2,166	2,779	2,096
MAF < 1% (SNPs)*	3,640,742	1,980,500	3,260,639
Call freq <95% (SNPs)*	1,729,493	1,704,412	1,766,376
Call rate < 95%, MDS outliers, IBD sharing (samples)*	0	0	0
Passing QC	729 (6,427,200)	959 (6,190,549)	502 (6,240,842)

Final number of samples (and SNPs in brackets) passing the genotype QC are reported in the bottom row. Note that these numbers do not also account for QC of the trait scores.

* After imputation QC. Before this step, imputed SNPs with *r*^2^ < 0.3 were filtered out, and all the genotypes with quality score <0.9 were set to missing.

† As *CLDRC-RD* and *CLDRC-ADHD* were processed together and drawn from the same population, we treated them as a single dataset in the genotype QC.

‡ As *UK-RD* samples had been genotyped on two different Illumina® platforms (see *Methods*) the subsets were analysed separately before imputation, and pre-imputation QC details are therefore reported for both the subsets (first genotyping wave with HumanHap 550k and second genotyping wave with Human OmniExpress). Note that 35 samples were genotyped on both of the arrays, and one of these samples showed inconsistent genotyping and was therefore discarded in both subsets.

§ The high number of SNPs discarded at this stage was due to the fact that no quality filter had been applied on this subset during genotype call process (see Appendix S4).

¶ In these cases, SNPs with call frequency <99% and samples with call rate <98% had already been discarded during genotype call process (see Appendix S4).

** Includes three sex chromosome abnormalities carriers.

†† Includes nine samples with sex chromosome abnormalities and one with X chromosome call rate <95%.

At the end of the genotype QC process, we had data for 959 participants and 6,190,549 polymorphisms in UK-RD, 729 participants and 6,427,000 polymorphisms in CLDRC, and 502 participants and 6,240,842 polymorphisms in SLIC, with 5,518,496 polymorphisms shared across all three datasets.

### Reading and language measures

Table2 lists the reading- and language-related traits that were assessed in the different datasets, as detailed in prior publications (Compton *et al.* 2001; Francks *et al.* 2004; Friend & Olson 2010; The SLI Consortium 2002, 2004). Further information on these measures is given in Tables5. To remove outliers, trait scores were excluded when they were more than three standard deviations from the relevant sample mean. Subjects with three or more such outliers were excluded from the dataset (one participant in UK-RD and one in CLDRC-RD). Reading/language traits had been previously age-adjusted according to normative data (Compton *et al.* 2001; Francks *et al.* 2004; Friend & Olson 2010; The SLI Consortium 2002, 2004). When a measure differed significantly from normality, we performed a within-dataset rank-normalization to attain normality and improve the suitability for principal components analysis (see Appendix S4 for details). We also excluded subjects showing full scale IQ < 70 (one participant from CLDRC-RD, and four participants from SLIC). This left 564 subjects in CLDRC-RD, 958 in UK-RD, 498 in SLIC and 163 in CLDRC-ADHD, which were used for the computation of the First Principal Component. Pairwise trait correlations within each dataset were calculated as the median correlation over 100 repeat random samplings of one individual from each independent sibship (see Appendix S4).

**Table 2:** Phenotypic traits available (when labelled by ‘x’) and measures used for PC1 derivation within each dataset (labelled with relative loadings on PC1 in parentheses)

Trait	Description (ability assessed)	CLDRC-RD (564)	UK-RD (958)	SLIC (498)	CLDRC-ADHD (163)
WRead	Reading real words	x (0.918)	x (0.918)	x (0.902)	x (0.871)
WSpell	Spelling real words	x (0.813)	x (0.852)	x (0.862)	x (0.764)
PD	Ability to convert letter strings into sounds, according to given phonetic rules	x (0.895, 0.861)*	x (0.809)		x (0.821, 0.729)*
PA	Ability to recognize and manipulate speech sounds (phonemes)	x (0.801)	x†		x (0.744)
OC	Ability to recognize a word as an orthographic unit and to retrieve the corresponding phonological form	x (0.764)	x (0.888)		x (0.644)
NWR	Ability to repeat nonsense words orally presented	x (0.493)		x (0.665)	x (0.355)
ELS	Sentence recalling and production (expressive domain of language)			x (0.856)	
RLS	Listening and auditory comprehension (receptive domain of language)			x (0.837)	
VIQ	Verbal reasoning	x	x	x	x
PIQ	Logical reasoning	x	x	x	x
PC1	Common variance in reading and language skills	544	914	245	159
IQ-adjusted PC1	Common variance in reading and language skills, not shared with general cognitive abilities	544	878	245	159

Sample sizes of the datasets (after genotype and phenotype QC) are reported in the header row. Sample sizes involved in the PC1 meta-analysis are reported at the bottom of the table (since we excluded participants with at least one missing measure among the traits involved in principal component analysis).

WRead, word reading; WSpell, word spelling; PD, phonological decoding; PA, phoneme awareness; OC, orthographic coding; NWR, nonword repetition; ELS/RLS, expressive/receptive language score; VIQ/PIQ, verbal/performance IQ.

* Loadings of nonword reading and phonological choice (respectively) on PC1s.

† Trait excluded from the PCA due to the low number of measures available.

**Table 3:** Language-/reading-related traits available in the UK-RD dataset

Trait	Test	Test description*	Statistical elaboration†
WRead	British Ability Scale (BAS)/Wide Range Achievement Test-Revised (WRAT-R)^1^,^2^	Reading aloud a series of real words presented on a card	A, S, R
WSpell	BAS/WRAT-R^1^,^2^	Writing words that are dictated by the test administrator	A, S, R
PD	Castles & Coltheart (C&C)^3^,^4^	Reading aloud nonsense words of increasing difficulty, according to English grapheme-phoneme conversion rules	A, S, R
	Nonword reading		
PA	Spoonerism test^5^,^6^	Simple phoneme deletion and substitution (e.g. replace the first sound in *dog* with *\l\* to make *log*)	A, S, R
		Complex phoneme deletion and substitution	
		Spoonerism (swapping the first sounds of two words, e.g. from *spoon, dog* to *doon*, *spog*)	
OC	C&C^3^,^4^	Reading aloud irregular words of increasing difficulty (i.e. words whose pronunciation does not follow the English grapheme-phoneme conversion rules, e.g. *yacht*)	A, S, R
	Irregular word reading		
vIQ	BAS/Wechsler Adult Intelligence Scale – Revised (WAIS-R)^7^	Similarities subtest only (explaining how two/three words are similar or go together)	A, S, R
pIQ	BAS^1^	Matrices subtest only (predicting missing components of increasingly complex matrices containing abstract symbols)	A, S

Superscript numbers after each test indicate the initial reference for it, where further details on the test can be found.

1 Elliot *et al.* 1979;

2 Jastak & Wilkinson 1984;

3 Castles & Coltheart 1993;

4 Coltheart & Leahy 1996;

5 Gallagher & Frederickson 1995;

6 Frederickson 1995;

7 Wechsler 1981.

* Where more than one battery is administered, the total score is usually computed as a sum of the raw scores from each subtest.

† Legend of trait adjustments: A, age-adjusted; S, standardized against the normative mean of the population of reference; R, further rank-normalized (using Blom's formula), because the trait distribution after standardization differed from normality (Shapiro–Wilk test *P* < 0.05).

**Table 4:** Language-/reading-related traits available in the SLIC dataset

Trait	Test	Test description*	Statistical elaboration†
WRead	Wechsler Objectives of Reading Dimensions (WORD)^1^	Reading single real words of increasing difficulty	A, S, R
WSpell	WORD^1^	Spelling of single real words	A
NWR	Gathercole & Baddeley^2^	Repeating tape-recorded nonsense words of increasing length and complexity	A, S, R
ELS	Clinical Evaluation of Language Fundamentals Revised (CELF-R)^3^	Formulating sentences (formulating sentences about visual stimuli using a targeted word or phrase)	A, S, R
		Recalling sentences (imitating sentences presented by the examiner)	
		Sentence assembly (producing two semantically/grammatically correct sentences from visually and orally presented words/groups of words)	
RLS	CELF-R^3^	Oral directions (pointing to pictured objects in response to oral directions)	A, S, R
		Semantic relations (listening to a sentence and selecting the two choices that answer a target question, out of four possible answers)	
		Word classes (choosing two related words and describing their relationship)	
vIQ	Wechsler Intelligence Scale for Children (WISC)/WAIS^4^	Arithmetic (solving orally administered arithmetic word problems)	A
		Comprehension (explaining situations, actions, or activities that the examinee is expected to be familiar with)	
		Digit span (reciting a sequence of digits presented by the examiner by recalling them in the same/reverse order)	
		Information (general cultural knowledge test)	
		Similarities (explaining how two words are alike/similar)	
		Vocabulary (defining a provided word)	
pIQ	WISC/WAIS^4^	Block design (arranging blocks to duplicate a given image/design)	A, S, R
		Coding (marking rows of shapes with different lines/transcribing symbols under digits, according to a given code)	
		Object assembly (correctly assembling the parts that an object is divided into, like a puzzle)	
		Picture arrangement (arranging a number of given pictures from left to right to tell the intended story)	
		Picture completion (identifying the missing part in a series of pictures representing common objects)	

Superscript numbers after each test indicate the initial reference for it, where further details on the test can be found:

1 Rust *et al.* 1993;

2 Gathercole *et al.* 1994;

3 Semel *et al.* 1992;

4 Wechsler *et al.* 1992.

* Where more than one battery is administered, the total score is usually computed as a sum of the raw scores from each subtest.

† Legend of statistical elaborations: A, age-adjusted; S, standardized against the normative mean of the population of study, when required (Shapiro–Wilk test *P* < 0.05); R, further rank-normalized (using Blom's formula), because the trait distribution after standardization differed from normality (Shapiro–Wilk test *P* < 0.05).

**Table 5:** Language-/reading-related traits available in the CLDRC dataset

Trait	Test	Test description*	Statistical elaboration†
WRead	Peabody Individual Achievement Test (PIAT)^1^	Reading aloud in sequence single real words increasing in semantic and phonetic difficulty, until errors are made in five out of any seven consecutive items (untimed)	C, A, S, R
	Timed oral reading^2^,^3^	Reading aloud a series of single real words within 2 seconds of their presentation, until errors are made in 10 out of any 20 consecutive items	
WSpell	PIAT^1^	Choosing the correct spelling of a series of real words (of increasing difficulty) orally presented, among four orthographically and often phonologically similar alternatives printed on a card (for each word), until errors are made in five out of seven consecutive responses	A, S
PD	Oral Nonword Reading Task^2^,^3^	Reading aloud a series of single-syllable nonsense words (structure ranging from *vcv* to *cccvcv*)	C, A, S, R
		Reading aloud a series of two-syllables nonsense words	
	Phonological Choice (Silent Nonword Reading Task)^2^,^3^	Choosing which of three nonsense words would sound like a real word if read aloud (for n triplets of nonwords)	A, S, R
PA	Phoneme Segmentation and Transposition Task^3^	Taking the first phoneme of a word, putting it at the end and add the sound*/ay/*(for n words, e.g. *rope* → *ope-ray*)	C, A, S, R
	Phoneme Deletion Task^3^	Repeating nonwords within 2 seconds of their oral presentation, then removing a specified phoneme and pronouncing the resulting words within another 4 seconds (e.g. ‘say *prot*..now say *prot* without the*/r/*’ ‘*pot*’)	
OC	Word-Pseudohomophone Choice^2^,^4^	Speeded forced-choice to distinguish a real word from a phonologically similar nonword (for *n* pairs of words-nonwords; e.g. rane vs. *rain*)	C, A, S, R
	Homophone Choice*,^4^	Selecting which of two homophones visually presented answers a question asked orally by the tester (for *n* pairs of words, e.g. ‘Which is a flower?’ *rose* rows)	
NWR	Gathercole & Baddeley^5^	Repeating tape-recorded nonsense words of increasing length and complexity	A, S, R
vIQ	WISC-R/WAIS-R^6^	Comprehension (explaining situations, actions, or activities that the examinee is expected to be familiar with)	None
		Information (general cultural knowledge test)	
		Similarities (explaining how two words are alike/similar)	
		Vocabulary (defining a provided word)	
pIQ	WISC-R/WAIS-R^6^	Block design (arranging blocks to duplicate a given image/design)	None
		Object assembly (correctly assembling the parts that an object is divided into, like a puzzle)	
		Picture arrangement (arranging a number of given pictures from left to right to tell the intended story)	
		Picture completion (identifying the missing part in a series of pictures representing common objects)	

Superscript numbers after each test indicate the initial reference for it, where further details on the test can be found:

1 Dunn & Markwardt 1970;

2 Olson *et al.* 1989;

3 Olson *et al.* 1994a;

4 Olson *et al.* 1994b;

5 Gathercole *et al.* 1994;

6 Wechsler 1974.

* Where more than one battery is administered, the total score is computed as a sum of the raw scores from each subtest (IQ measures), as an average of *z*-scores derived from accuracy scores (% of correct responses) and median correct reaction times of the two subtests (nonword reading), or as the arithmetic average of the raw scores from each subtest (all the other measures).

† Legend of statistical elaborations: C, composite score; A, age-adjusted (score regressed against age and age^2^); S, standardized against the normative mean of a control population; R, further rank-normalized (using Blom's formula), because the trait distribution after standardization differed from normality (Shapiro–Wilk test *P* value < 0.05).

#### First Principal Component score computation

The First Principal Component from all of the language- and reading-related traits available (PC1, Table2) was derived in each dataset, through the SPSS® 20.0 Factor Analysis (Principal Component extraction method, hereafter called PCA). This reduced our correlated measures into a smaller set of latent variables (factors or principal components) that can explain the maximum amount of shared variance (Field 2005). In each dataset, only linear components with Eigenvalue >1 were extracted, allowing for correlation among the components (oblique rotation, *direct oblim* method) and excluding subjects with any missing measure (*missing listwise* option). A Kaiser–Meyer–Olkin measure of sampling adequacy and a Bartlett's test of sphericity were run in all the PCAs. These tests revealed a high common variance (KMO = 0.8–0.9) and a significant interdependence (Bartlett's test *P* value < 0.05) among the variables examined in each dataset, justifying the PCAs.

The proportion of total variance explained by PC1 was 75.3% in UK-RD, 68.6% in SLIC, 64.5% in CLDRC-RD and 52.0% in CLDRC-ADHD. In all the datasets PC2 explained no more than 13% of the total variance. All of the PC1s showed a broad pattern of loadings across the traits (Table2). The total number of participants for which we finally obtained genotype and PC1 data (i.e. all datasets combined) was 1862. We also obtained residuals from regressing PC1 against performance IQ (which had not been included in PC1 computation), again separately within each dataset. A measure of performance IQ was not available for 36 of the 1862 participants, and therefore the total sample size for IQ-adjusted PC1 was 1826.

We also derived a first principal component score within each dataset from only word reading and spelling, because these were the only measures available in all datasets and therefore provided a possibility to match traits as closely as possible across datasets. The first PC derived from word reading and spelling is referred to as PC1_read_ hereafter. The proportion of variance in word reading and spelling explained by PC1_read_ was 86.9% in UK-RD, 88% in CLDRC-RD, 93.4% in SLIC and 80.1% in CLDRC-ADHD. As only two measures were used to construct PC1_read_ then the measures loaded equally onto this component, and the loadings were high in all datasets (≥0.9). PC1_read_ was therefore a highly comparable construct across datasets (see Appendix S3). Moreover, the correlation between PC1 and PC1_read_ was high in each dataset (Pearson's *r* = 0.925 in CLDRC-RD, 0.947 in UK-RD, 0.914 in SLIC and 0.917 in CLDRC-ADHD), so that PC1 itself could also be regarded as highly comparable across datasets. Note that these correlations were based on repeat random sampling of one member from each unrelated sibship (as for all pairwise trait correlations; see above). The total number of subjects across all datasets for PC1_read_ was 1913, and for IQ-adjusted PC1_read_ it was 1875. We primarily focused on PC1 for our subsequent genetic analysis (below), because this would maximize the chance of identifying SNPs that affect variance shared between both reading and language measures. However, we also repeated GWAS meta-analysis using PC1_read_ to provide a comparable analysis that would be minimally affected by the heterogeneity of available measures across datasets.

### Genetic association analyses

#### Sibling-pair GWAS

Sibling-based genome-wide association analyses were conducted using PC1 and PC1_read_ scores separately within each dataset, both before and after IQ-adjustment, and using the ‘total’ association option of the QFAM function implemented in PLINK v1.07 (http://pngu.mgh.harvard.edu/∼purcell/plink/; Purcell *et al.* 2007). This method tests for association at each SNP by regressing trait scores on genotypes in an additive linear model. To correct for non-independence of siblings, permutations were run (i.e. label-swapping of phenotypes/genotypes) to obtain empirical significance levels (further details in Appendix S4).

#### GWAS meta-analysis (GWASMA)

The results from GWAS in the separate datasets were then meta-analysed together. This was implemented in the programme METAL (http://www.sph.umich.edu/csg/abecasis/Metal/index.html; Willer *et al.* 2010). We chose an approach that does not assume equivalence of allelic effect sizes between datasets, which was appropriate given the heterogeneity of study recruitment and assessment. Put briefly, the GWAS meta-analysis tested each SNP for a genetic effect, across the contributing datasets, computing an overall *z*-score for that SNP determined by the *P* value, the direction of the allelic effect on the quantitative trait, and the sample size of each study involved in the meta-analysis.

#### Gene-based analysis

The results of the GWASMA on PC1 were used as input for gene-based association analyses using VEGAS v0.8.27 (http://gump.qimr.edu.au/VEGAS/; Liu *et al.* 2010). This software performs association tests for ∼18,000 autosomal genes, by assigning multiple SNPs to each individual gene according to their genomic locations, and then combining the evidence for association across all SNPs assigned to a given gene, while taking into account the linkage disequilibrium (LD) structure between SNPs. Each tested gene also included potentially regulatory regions located up to 50 kb beyond the 5′- and 3′-untranslated regions (UTRs). A Bonferroni-corrected significance threshold was set at *P* < 2.8 × 10^−6^ to account for the number of genes tested (see Appendix S4 for details).

#### Pathway-based analysis

Finally, a pathway/network-based association analysis was run using the PC1 GWASMA results, with the programme INRICH v1.0 (http://atgu.mgh.harvard.edu/inrich/started.html; Lee *et al.* 2012). This tool tests for an enrichment of association within predefined gene sets, through a permutation-based approach. We defined associated genomic intervals as those containing an individual association *P* < 0.001 in the GWASMA results. Gene boundaries were again defined as extending 50 kb beyond the 5′- and 3′-UTRs. Three candidate gene lists, based on the gene sets of the Gene Ontology Database (http://www.geneontology.org/), were tested for an enrichment of association. These represented three distinct neurobiological hypotheses on the aetiology of reading and language disabilities: axon guidance (including all the GO sets containing the term ‘axon guidance’), neuronal migration (including all the GO sets containing the term ‘neuron migration’) and steroid sex hormone biology (including all the GO sets containing the terms ‘steroid’, ‘androgen’, ‘oestrogen’, ‘progesterone’ and ‘testosterone’). Further details on the analysis can be found in Appendix S4.

### Further analysis of top association signals

#### Effect sizes on different traits

We repeated the regressions of PC1 and IQ-adjusted PC1 on the genotypes of our two most significantly associated SNPs from GWAS meta-analysis, in an additive linear model, in order to conveniently obtain the regression *r* ^2^ as indicative measures of effect sizes. To generate measures unbiased by sample relatedness, regression *r* ^2^ were calculated in R (R Core Team 2013, http://www.r-project.org/) as the median *r* ^2^ over 100 repeat random samplings of one individual from each independent sibship, separately in each dataset.

We further investigated each of our top two association signals by running QFAM univariate association tests in PLINK v1.07 (Purcell *et al.* 2007) for each individual trait that was used in constructing PC1, and separately in each dataset. This analysis provided an initial assessment of pleiotropy for these loci. We also performed multivariate association analysis for these two loci, in PLINK Multivariate v1.06 (https://genepi.qimr.edu.au/staff/manuelF/multivariate/main.html; Ferreira & Purcell 2009), again separately in each dataset and using each of the reading/language traits that were used in constructing PC1. PLINK multivariate extracts the linear combination of traits that explains the largest possible amount of covariance between the SNP and all of the traits. The loading produced for each trait represent its contribution to the multivariate association. MQFAM ‘total’ association was run, with adaptive permutations to adjust for sample relatedness (see Appendix S4 for details).

#### Assessment of top association signals in two additional datasets

Our two most significant association signals from PC1 meta-analysis were checked against published and unpublished results from the recent GWASMA of reading and language abilities reported by Luciano *et al.* (2013). This prior study analysed two population datasets, the Brisbane Adolescent Twin Sample (*BATS*) and the Avon Longitudinal Study of Parents and their Children (*ALSPAC*). *BATS* is a cohort of twins and their non-twin siblings recruited from ongoing studies of melanoma risk factors and cognition in an Australian population-based sample (Wright *et al.* 2001). Subjects had been administered psychometric tests assessing regular-, irregular-, and nonword reading, and spelling, together with the Schonell graded word reading test, and nonword repetition (see Luciano *et al.* 2013). *ALSPAC* is a longitudinal, population-based sample recruited from the county of Avon, UK (Boyd *et al.* 2013). The study website contains details of all the data available through a fully searchable data dictionary (http://www.bris.ac.uk/alspac/researchers/data-access/data-dictionary). Ethical approval was obtained from the ALSPAC Ethics and Law Committee and the Local Research Ethics Committees. Participants (all free of neurological/psychiatric conditions) had been tested for word reading, nonword reading, spelling and nonword repetition (see Luciano *et al.* 2013). BATS and ALSPAC had been genotyped using Illumina® 610k Quad Bead and HumanHap 550k Quad chips, respectively, and imputed using the HapMap Phase II CEU reference panel (NCBI build 36) (The International HapMap 3 Consortium, 2010). A total of 6434 subjects (962 from BATS and 5472 from ALSPAC) were meta-analysed by Luciano *et al.* (2013), for three different traits: word reading, nonword repetition and a composite/component score of reading and spelling (called hereafter the *reading–spelling factor*).

## Results

### GWAS meta-analysis

**Figure 1:**
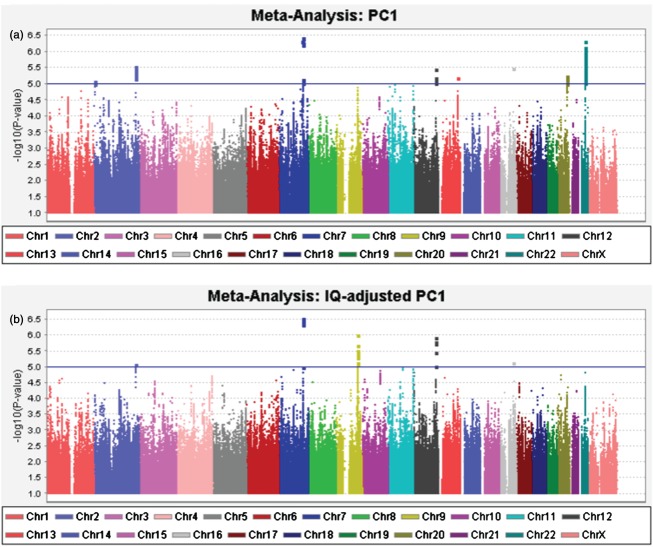
Manhattan plots of the meta-analyses of the first principal component scores. (a) PC1; (b) IQ-adjusted PC1. The blue line represents the nominal suggestive significance threshold (*P* = 1 × 10^–5^).

Table6 describes the most significant associations from the meta-analyses on PC1 (*N* = 1862) and IQ-adjusted PC1 (*N* = 1826). Figure 1 shows genome-wide Manhattan Plots. QQ-plots revealed no evidence of population stratification affecting the meta-analysis results, or of genome-wide significant associations [Fig. S1a,b (Appendix S1)]. The most significant association was observed for rs59197085 in PC1 and IQ-adjusted PC1 meta-analyses (*P* = 3.86 × 10^–7^ for PC1, and *P* = 3.01 × 10^–7^ for IQ-adjusted PC1; A/G, MAF ∼ 8%). This SNP is located at 7q32.1, within *CCDC136* (coiled-coil domain containing 136, or *NAG6*) and ∼10 kb upstream of *FLNC* [filamin C; Fig. S1c (Appendix S1)]. The second most significantly associated region, before IQ-adjustment, was located on 22q12.3, SNP rs5995177 (*P* = 5.01 × 10^–7^, A/G, MAF ∼ 8%), within *RBFOX2* [also known as RNA-binding motif protein 9, or *RBM9*; Fig. S1d (Appendix S1)]. The association was less significant after IQ-adjustment of PC1 (*P* = 1.5 × 10^–5^), and this difference was not merely due to the loss of 36 subjects in the IQ-adjusted analysis (investigated by performing a repeat PC1 analysis in the same reduced set of subjects as were available for IQ-adjusted PC1, data not shown).

**Table 6:** Top association signals (*P* < 1 × 10^–6^) in the PC1 and IQ-adjusted PC1 meta-analyses

Chr	SNP*	Position (hg19)	Allele1	Allele2	Freq Allele1 (%)	*P* value	Direction†	Gene (distance)‡	Variant type
*PC1*									
7	rs59197085	128460756	a	g	7.971	3.86 × 10^–7^	– – – –	FLNC(–9.726)|CCDC136(0)	Intronic
7	rs58845495	128462847	t	c	92.029	4.09 × 10^–7^	+ + + +	FLNC(–7.635)|CCDC136(+0.664)	
7	7:128439695:I	128439695	i	r	7.94	4.99 × 10^–7^	– – – –	CCDC136(0)	Intronic
22	rs5995177	36309553	a	g	8.049	5.01 × 10^–7^	– – – –	RBFOX2(0)	Intronic
7	rs3734972	128470838	t	c	7.983	5.66 × 10^–7^	– – – –	FLNC(0)|CCDC136(+8.655)	Exonic,synonymous
7	rs3800560	128461094	t	c	7.971	6.25 × 10^–7^	– – – –	FLNC(–9.388)|CCDC136(0)	Intronic
22	rs12158565	36316843	c	g	87.23	7.57 × 10^–7^	+ + + +	RBFOX2(0)	Intronic
22	rs5755979	36290707	t	c	12.77	9.05 × 10^–7^	– – – –	RBFOX2(0)	Intronic
22	rs5750202	36339542	t	c	12.77	9.06 × 10^–7^	– – – –	RBFOX2(0)	Intronic
22	rs5750203	36339998	a	t	87.23	9.72 × 10^–7^	+ + + +	RBFOX2(0)	Intronic
*IQ-adjusted PC1*									
7	rs59197085	128460756	a	g	7.971	3.01 × 10^–7^	– – + –	FLNC(–9.726)|CCDC136(0)	Intronic
7	rs58845495	128462847	t	c	92.029	3.23 × 10^–7^	+ + – +	FLNC(–7.635)|CCDC136(+0.664)	
7	rs3800560	128461094	t	c	7.971	3.95 × 10^–7^	– – + –	FLNC(–9.388)|CCDC136(0)	Intronic
7	7:128439695:I	128439695	i	r	7.94	4.48 × 10^–7^	– – + –	CCDC136(0)	Intronic
7	rs3734972	128470838	t	c	7.983	4.68 × 10^–7^	– – + –	FLNC(0)|CCDC136(+8.655)	Exonic,synonymous

* Single-base indels were not filtered out from the imputed polymorphisms since they were reliably called in the imputation reference (1000 Genomes, Phase I v3), and were tested for association as they could represent coding frameshift variants of biological interest.

† The direction of effect of Allele1 is reported for datasets in the following order: CLDRC-RD, UK-RD, SLIC, CLDRC-ADHD.

‡ Physical distance (kb) from closest genes (in a ±10 kb range from each marker) is indicated, along with orientation based on the direction of transcription (‘–’, upstream of 5'-UTR; ‘+’, downstream of 3′-UTR).

Table S2a,b (Appendix S2) shows all SNPs with association *P* < 1 × 10^–5^ in GWAS meta-analysis of PC1 or IQ-adjusted PC1. No genome-wide significant associations were observed in the GWAS in the individual datasets (data not shown).

The results of our complementary PC1_read_ meta-analysis (Appendix S3) were consistent with the PC1 meta-analysis, with rs59197085 and rs5995177 among the top suggestive associations (*P* ∼ 10^–6^). This was expected given the high correlations between PC1 and PC1_read_ in each dataset (all correlations >0.9, see above).

### Effect sizes and profiles of top associations

rs59197085 (*CCDC136*/*FLNC*) explained 3% of PC1 variance and 3.2% of IQ-adjusted PC1 variance in our largest GWAS dataset (UK-RD), and 1.3% of PC1 variance and 1.5% of IQ-adjusted PC1 variance in the next largest dataset (CLDRC-RD). The estimated effect sizes in the smaller datasets were ≤0.2%. Estimated effect sizes for rs5995177 (*RBFOX2*) were more consistent across datasets. This SNP explained 1.2% of PC1 and IQ-adjusted PC1 variance in UK-RD, and 1.8% of PC1 variance and 1.2% of IQ-adjusted PC1 variance in CLDRC-RD, while estimated effect sizes in the smaller datasets were between 0.6% and 1.6% of variance.

Both rs59197085 and rs5995177 showed broad profiles of association across the measures that were used to construct PC1, as assessed from the PLINK multivariate loadings and corresponding QFAM univariate association *P* values shown in Table7. These findings suggest pleiotropic effects of the two SNPs on reading and language.

**Table 7:** Effect of the top association signals rs59197085 (7q32.1) and rs5995177 (22q12.3) on the single reading and language traits used in constructing PC1

Trait	CLDRC-RD	UK-RD	SLIC	CLDRC-ADHD
rs59197085				
WRead	–0.66 (0.024)	–0.87 (5.3 × 10^–5^)	–0.29 (0.626)	–0.5 (0.427)
WSpell	–0.89 (3.8 × 10^–3^)	–0.75 (1.1 × 10^–3^)	0.08 (0.862)	–0.1 (0.871)
PD	–0.76 (7.9 × 10^–3^), –0.50 (0.081)*	–0.86 (1.6 × 10^–5^)		–0.37 (0.549), 0.13 (0.854)*
PA	–0.65 (0.029)	–0.49 (0.018)†		0.35 (0.588)
OC	–0.64 (0.036)	–0.89 (3 × 10^–6^)		–0.04 (0.95)
NWR	–0.34 (0.269)		–0.57 (0.32)	–0.28 (0.686)
ELS			–0.25 (0.807)	
RLS			0.08 (0.821)	
rs5995177				
WRead	–0.66 (0.027)	–0.81 (2 × 10^–3^)	–0.71 (0.116)	0.01 (0.98)
WSpell	–0.81 (6.9 × 10^–3^)	–0.82 (1.1 × 10^–3^)	–0.52 (0.262)	–0.33 (0.359)
PD	–0.65 (0.026), –0.79 (8.9 × 10^–3^)*	–0.77 (1.8 × 10^–3^)		–0.46 (0.158), –0.37 (0.26)*
				
PA	–0.72 (0.023)	–0.72 (2.5 × 10^–3^)†		–0.65 (0.046)
OC	–0.68 (0.026)	–0.57 (0.017)		–0.02 (0.968)
NWR	–0.04 (0.922)		–0.23 (0.674)	0.06 (0.876)
ELS			–0.82 (0.057)	
RLS			–0.61 (0.206)	

These were computed for each trait as PLINK Multivariate MQFAM loadings and PLINK univariate QFAM association *P* values (in brackets) and refer to the minor alleles (A for both SNPs).

WRead, word reading; WSpell, word spelling; PD, phonological decoding; PA, phoneme awareness; OC, orthographic coding; NWR, nonword repetition; ELS/RLS, expressive/receptive language score.

* Loading on nonword reading and phonological choice (respectively).

† Although PA had been excluded from the PCA in UK-RD (due to the low number of measures available), it was tested in this case to have a term of comparison to the other datasets.

### Gene-based meta-analysis

The strongest gene-based associations inferred from the PC1 and IQ-adjusted PC1 meta-analyses are reported in Table S2c,d (Appendix S2). While no gene exceeded the appropriate genome-wide significance threshold for this analysis (*P* < 2.8 × 10^−6^), *CCDC136*, *FLNC* and *RBFOX2* were among the most significantly associated genes, with the latter approaching the significance threshold in the PC1 analysis (*P* = 5 × 10^–6^). However, after conditioning on the most significant association signal within each gene, no other SNP within each of these genes showed significant evidence for having an independent residual effect, after correction for multiple testing [Table S2e,f (Appendix S2)]. For this analysis the gene boundaries were defined in the same way as for gene-based analysis (see above).

### Pathway-based meta-analysis

We assessed evidence for an excess of association signals within the genes of three neurobiological pathways that are prominent in prior literature on reading and language: axon guidance, neuronal migration and steroid sex hormone biology (see *Discussion* for the relevant citations). None of the three tested sets showed significant associations with PC1 or IQ-adjusted PC1 [Table S2g,h (Appendix S2)], although the association between PC1 and the steroid-related pathway approached significance (*P* = 0.051).

### Assessment of top associations within previous GWAS results

We assessed our most significant associations from PC1 meta-analyses within published and unpublished results from the previous GWAS study of the *BATS/ALSPAC* datasets, for which the reading and language measures were IQ-adjusted (Luciano *et al.* 2013). *FLNC* and *CCDC136* showed nominally significant associations in gene-based (VEGAS) analyses of reading-related traits in *BATS/ALSPAC* (*CCDC136 P* = 0.034 for reading-spelling factor and *P* = 0.003 for word-reading; *FLNC P* = 0.009 for word- reading; see Table S3 of Luciano *et al.* 2013). The reading–spelling factor in the *BATS*/*ALSPAC* datasets was the most comparable trait to the IQ-adjusted PC1 score of this study. As the study of Luciano *et al.* 2013 had used the HapMap2 reference dataset for genotype imputation, it was not possible to directly investigate the most highly associated SNPs from this study in the *BATS/ALSPAC* datasets. We therefore investigated association for two HapMap2 SNPs that were closest to our top hits on 7q32 and 22q12.3. rs3734972 (PC1 *P* = 5.66 × 10^–7^, IQ-adjusted PC1 *P* = 4.68 × 10^–7^; T/C, minor allele T, MAF ≈ 8%) lies ∼10 kb away from rs59197085 on 7q32 and is in high LD with it [*R* ^2^ = 0.89, see local association plot, Fig. S1c (Appendix S1)]. rs3734972 showed a *P* value of 0.032 with the IQ-adjusted reading-spelling factor in *BATS/ALSPAC*. The allelic trend was in the opposite direction to that observed in the *UK-RD/SLIC/CLDRC* datasets, with the T allele having a positive effect on the trait score in the *BATS/ALSPAC* cohorts. rs12158565 (PC1 *P* = 7.57 × 10^–7^, IQ-adjusted PC1 *P* = 4.65 × 10^–5^; C/G, minor allele G, MAF ≈ 13%) was the second most significant association in 22q12.3, mapping ∼7 kb from the top SNP at this locus rs5995177, and in low LD with it (*R* ^2^ = 0.083), as are all the other suggestively associated SNPs in 22q12.3 [see local association plot, Fig. S1d (Appendix S1)]. rs12158565 showed no evidence of association in *BATS/ALSPAC* (*P* = 0.81).

## Discussion

This study aimed to identify pleiotropic variants having effects on reading and language abilities by analysing continuous traits in multiple datasets. Our study is complementary to two recently published GWAS: one using a similar approach but in general population samples (Luciano *et al.* 2013), and another contrasting a relatively small number of categorically defined RD-SLI comorbid cases with unaffected controls (Eicher *et al.* 2013).

Our study is novel and distinct for several reasons:

First, we analysed continuous variation in reading and language skills while also having an enrichment of participants with low abilities (i.e. through analysing poor performing probands together with their siblings), and without applying a dichotomous classification into cases and controls that necessarily involves arbitrary thresholding. Our design was therefore suited to detect genetic effects on susceptibility to RD and SLI that also act across the entire distribution of reading and language skills.

Second, we specifically focused on shared neurobiological mechanisms underlying language and reading, by analysing the first principal component of all of the reading- and language-related measures available in each dataset, followed by investigating the cross-phenotypic effects of the resulting top GWAS hits through univariate association analysis using each individual measure. We additionally followed this with a confirmatory analysis focused only on word reading and spelling, because these measures provided the closest matching possibility across our datasets. The first principal component (PC1) of all available measures extracted a large proportion of shared trait variance across the two domains of reading and language, and was highly correlated with the component derived from only reading and spelling (PC1_read_).

Third, we performed GWAS both before and after IQ-adjustment of PC1. This was done in order to identify both genetic variants having effects broadly across reading, language and general cognitive abilities, and variants having effects on reading and language but independently of general cognitive ability. This approach also facilitated a comparison of our top results with those from datasets investigated in Luciano *et al.* (2013).

We checked within our GWASMA results 18 specific SNPs that had been highlighted to show the most promising candidate associations by the authors of previous GWAS studies of reading and/or language (Eicher *et al.* 2013; Field *et al.* 2013; Luciano *et al.* 2013; Meaburn *et al.* 2008; Roeske *et al.* 2011). Seventeen of these SNPS showed no nominally significant association within our GWASMA results (data not shown). Only rs10485609 (Meaburn *et al.* 2008) showed a nominally significant association (*P* = 0.013 for PC1, *P* = 0.015 for IQ-adjusted PC1; allele A was associated with lower performance, which was a consistent allelic direction of effect with that reported by Meaburn *et al.* 2008), but this was not significant after multiple testing correction for 18 tests.

Like the other recently published GWAS efforts in this field, our study did not find any individual associations that achieved genome-wide significance (threshold *P* = 5 × 10^–8^). However, we did identify two novel, suggestive results of particular interest, on 7q32.1 and 22q12.3, with the most significant associations at rs59197085 and rs5995177, respectively. As shown in Table7, both SNPs displayed a broad pattern of association across multiple reading and language traits, consistent with effects on neurobiological processes shared between reading and language cognition. In the regression model these SNPs explained a notable proportion (up to 3.2%) of variance in PC1 and IQ-adjusted PC1 scores, particularly in the largest datasets (CLDRC-RD and UK-RD), although these effect sizes are likely to be overestimated since this is the first report of these associations (Ioannidis 2008). Gene based-tests were consistent with the results of the SNP-based analysis for *FLNC*, *CCDC136* and *RBFOX2*, and the gene-based *P* values were found to be largely or wholly reflective of the individual top associations within each of these genes.

rs5995177 is an intronic variant localized within *RBFOX2* (RNA-binding protein, fox-1 homologue 2, also known as *RBM9*), a protein that regulates alternative splicing and is active in neurons. *RBFOX2* is highly expressed in the foetal brain and has important roles in CNS development (Gehman *et al.* 2012). The homologous gene *RBFOX1* has been implicated in several neurodevelopmental disorders, including Rolandic Epilepsy (Lal *et al.* 2013) and Autism Spectrum Disorder (Voineagu *et al.* 2011), and is a downstream target of FOXP2, a transcription factor implicated in monogenic speech and language disorders (Ayub *et al.* 2013). The high comorbidity between Rolandic Epilepsy and RD (Clarke *et al.* 2007) and the presence of a FOXP2 binding site ∼5 kb from rs5995177 (The ENCODE Project Consortium, 2012), further support a link of *RBFOX2* with reading and language abilities. Thus convergent evidence from multiple lines of research makes *RBFOX2* an intriguing candidate gene for future studies. There was no evidence of association of this locus with reading and language measures in the results of the population-based study of Luciano *et al.* (2013).

rs59197085 is located in *CCDC136* (coiled-coil domain containing 136, or *NAG6*) and ∼10 kb upstream of *FLNC* (filamin C). This SNP, along with the nearby SNPs rs3800560, rs58845495 and rs3734972, forms roughly 10-kb haplotypes spanning the region between *CCDC136* and *FLNC* and partially overlapping these genes [see local association plot, Fig. S1c (Appendix S1)]. *CCDC136* encodes a poorly characterized tumour suppressor which has been found to be downregulated in gastric carcinoma (Zhang *et al.* 2004) and is highly expressed in the cerebellum and in the occipital cortex (Allen Human Brain Atlas, Hawrylycz *et al.* 2012; http://human.brain-map.org). Filamin C (or filamin gamma) is a structural protein that crosslinks actin filaments into orthogonal networks in the cortical cytoplasm and participates in cytoskeleton re-modelling, suggesting a possible role in cell motility and migration. Functions of *FLNC* have been demonstrated in muscle tissues, where mutations are responsible for several forms of myopathies (Duff *et al.* 2011). However, its pattern of expression includes spinal cord, cerebellum, corpus callosum, basal ganglia and some localized areas in the frontal, temporal and occipital cortex (Allen Human Brain Atlas, Hawrylycz *et al.* 2012). Its homologue *FLNA* (filamin A) is involved in neuronal migration and is implicated in an X-linked dominant form of periventricular heterotopia, a neurological disorder that sometimes involves reading and spelling problems (Robertson 2005).

Associations within the 7q32 region are particularly interesting in light of data from two previous independent studies that have each reported evidence for linkage between a microsatellite marker in this region (D7S530, located ∼650 kb from our peaks of association) and RD status (Kaminen *et al.* 2003) or else nonword spelling and irregular word reading (Bates *et al.* 2007). There was also evidence of association, at the gene level, with reading and language measures for *FLNC*, and *CCDC136* in the *BATS/ALSPAC* datasets studied by Luciano *et al.* (2013). At the SNP level, one of our most significantly associated SNPs from GWASMA, rs3734972 also showed association with an IQ-adjusted reading–spelling score in the *BATS*/*ALSPAC* datasets. However, the allelic directions of effect on the traits in this study and the study by Luciano *et al.* were opposite.

We sought to detect an excess of association signals within genes belonging to each of three candidate gene sets based on different biological functions: axon guidance, neuronal migration and steroid hormone biology. Axon guidance and neuronal migration are functions linked to some of the previously identified candidate genes in RD and SLI; *ROBO1* (Hannula-Jouppi *et al.* 2005), *DCDC2* (Meng *et al.* 2005), *KIAA0319* (Peschansky *et al.* 2010), *DYX1C1* (Tammimies *et al.* 2013) and *FOXP2* (Vernes *et al.* 2011). A potential involvement of neuronal migration deficits in RD aetiology represents a longstanding hypothesis of the field (see Galaburda & Cestnick 2003). The steroid hypothesis was motivated by literature suggesting links between sex hormone biology, language performance and the brain architecture that subserves reading and language (Good *et al.* 2001; Lombardo *et al.* 2012; Shapleske *et al.* 1999; Whitehouse *et al.* 2012); and by evidence of interaction between Oestrogen Receptors and DYX1C1, both at the gene (Tammimies *et al.* 2012) and at the protein level (Massinen *et al.* 2009). None of the three gene sets showed a significant excess of association signals, although the steroid hormone biology set approached significance in this analysis.

In carrying out GWASMA studies of complex cognitive traits across multiple datasets collected by different research teams, an obvious limitation is that the specific trait measurements that are available may be quite diverse. Even when tests are similar, and hypothesized to measure corresponding cognitive processes, they may still create a substantial source of heterogeneity for a meta-analysis effort. In this study we sought to overcome this limitation by focusing on a principal component (PC1) capturing a majority of the shared variance between reading- and language-related traits. In spite of the phenotypic heterogeneity of our datasets, this measure can be considered comparable across datasets for a number of reasons. First, the loadings of the individual traits on PC1 scores were similar across the datasets. Second, dropping one or more traits from our PC1 computation did not substantially affect the resulting PC1 scores (data not shown). Third, the First Principal Component derived only from word reading and spelling (PC1_read_) was strongly correlated with PC1. Word reading and spelling were the only two measures available in all of the datasets and provided the closest phenotype matching possible across datasets. Not surprisingly, given the high correlations between PC1 and PC1_read_ in all datasets, the association meta-analysis using PC1_read_ (Appendix S3) produced results consistent with PC1-based meta-analysis. We therefore conclude that PC1 was a sufficiently well matched construct across datasets to support GWASMA, in which we nonetheless allowed for heterogeneity of effect sizes across datasets to avoid assuming a perfect matching. It is interesting that a single PC can capture comparable variation across a diverse range of reading and language traits and in the presence of heterogeneity of measurement across datasets. This indicates a robust unifying dimension to much of this variation, and supports a genetic approach framed around pleiotropy.

The use of a principal component can lead to some loss of information, both in terms of detecting trait-specific genetic effects, and of reducing the sample size (because individuals with one or more missing trait values were excluded from the analysis). However, as we aimed to identify shared genetic effects on reading and language, the use of PC1 scores, followed by investigating cross-phenotypic associations of the top SNPs at the level of individual traits, was an appropriate approach to analysing these multivariate datasets. There is now a need for a larger international meta-analysis effort that incorporates further datasets. This would improve the power to detect pleiotropic variants affecting reading and language.

## Supporting Information

**Appendix S1**: QQ plots and association plots of the top association signals from analysis based on PC1 and IQ-adjusted PC1. Contribution of each dataset to the strength of the association in the PC1 and IQ-adjusted PC1 meta-analysis, for the top association signals.

**Appendix S2**: SNP-, gene- and pathway-based meta-analysis results from analysis based on PC1 and IQ-adjusted PC1. Test for independent associations within the top associated regions, after conditioning on the most significant local association signal.

**Appendix S3**: Analyses based on PC1_read_ and IQ-adjusted PC1_read_ (trait loadings on PC1_read_, Manhattan plots, QQ plots and top associations)

**Appendix S4**: Genotype calls and QC protocols; correlation patterns of reading and language measures; statistical analyses, commands and parameters.
