# Supplementary material for: Genome-wide screening for DNA variants associated with reading and language traits
Source: Genes Brain Behav. 2014 Aug 29;13(7):686–701. doi: 10.1111/gbb.12158 (PMC4165772; doi:10.1111/gbb.12158)
Supplement: Supplementary file 1 — Appendix S1: QQ plots and association plots of the top association signals from analysis based on PC1 and IQ-adjusted PC1. Contribution of each dataset to the strength of the association in the PC1 and IQ-adjusted PC1 meta-analysis, for the top association signals. [file gbb0013-0686-sd1.docx]

***Supplementary Material S1***

**QQ plots.**

a)


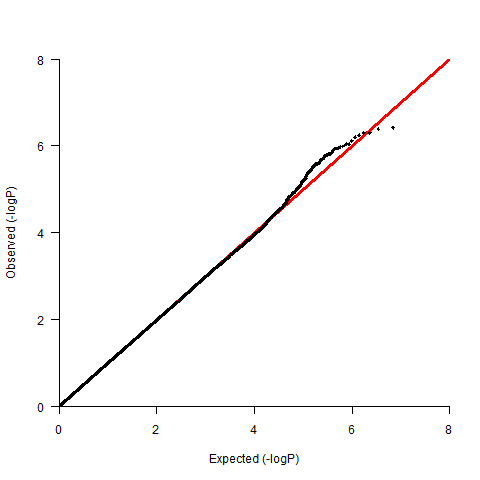


b)


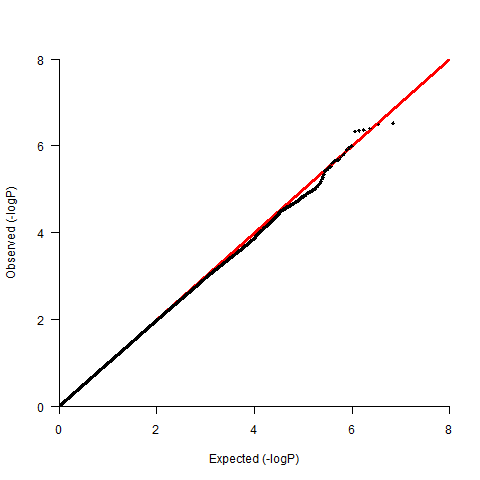


**Figure S1 a, b.** QQ-plots of the **a)** PC1 and **b)** IQ-adjusted PC1 meta-analyses. The plots were drawn through a dedicated R script (R Core Team, 2013, <http://www.r-project.org/>).

**Association plots.**

c)


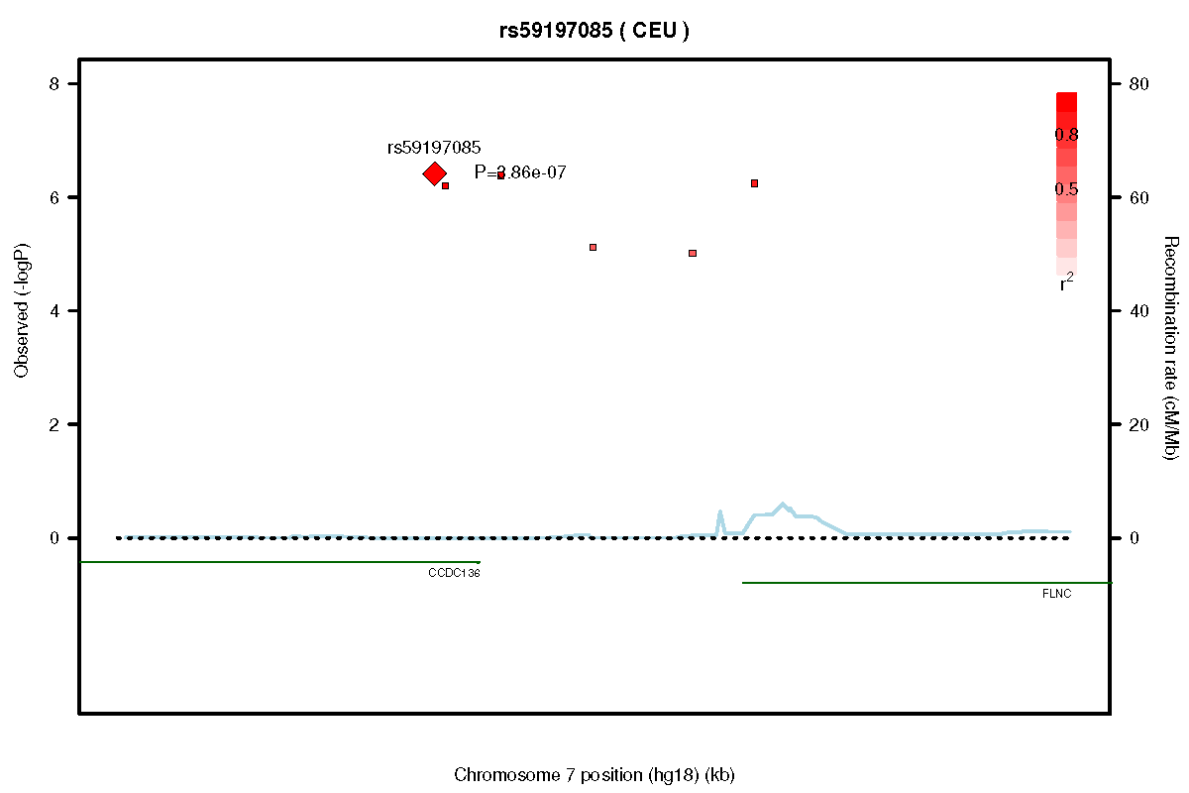


d)


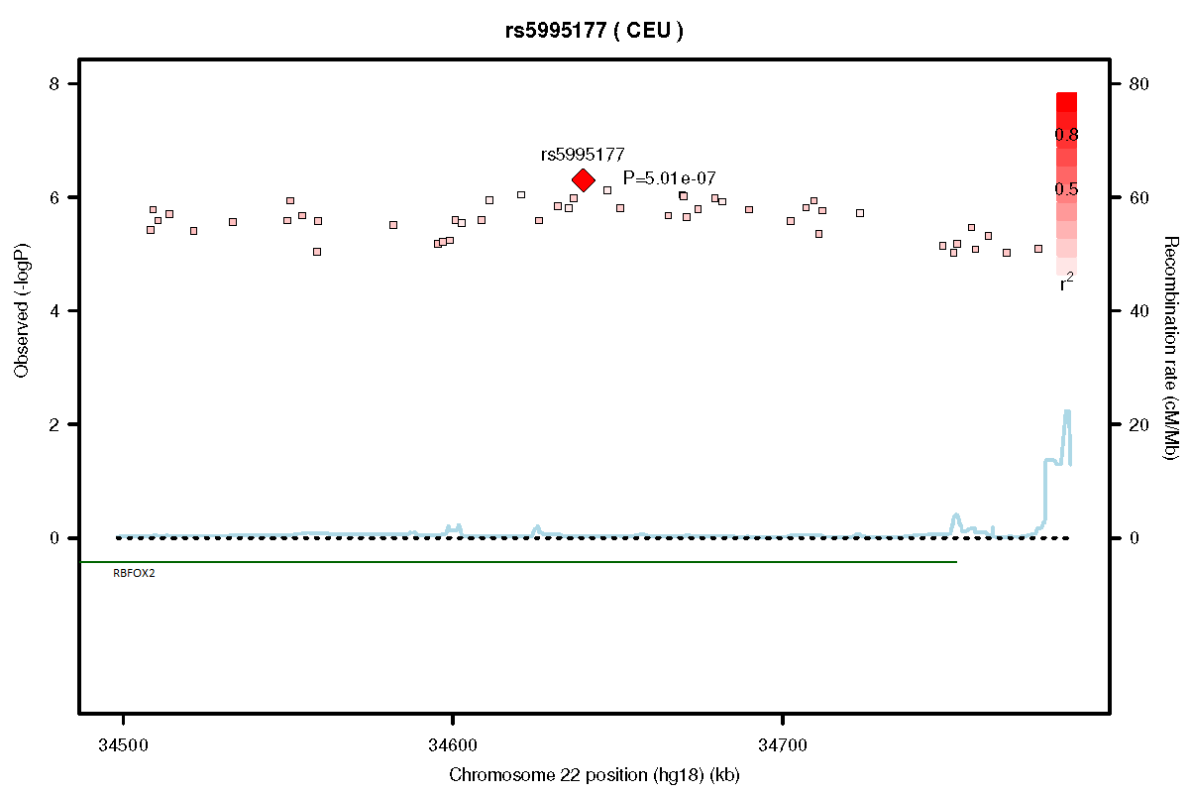


**Figure S1 c, d.** Association plots of the suggestive hits on **c)** 7q32.1 and **d)** 22q12.3 in the PC1 meta-analysis. All the suggestively associated SNPs (p < 1x10^-5^) are shown. Each squared dot represents a suggestively associated SNP in the region and the intensity of color fill represents the level of LD (r^2^) with the local top hit (light red indicates low LD, dark red indicates high LD).Note: the plots were produced through the SNAP tool (Johnson et al., 2008; <http://www.broadinstitute.org/mpg/snap/ldsearch.php>). However, Figure d was slightly modified in order to represent the isoform 5 of *RBFOX2* (the same used for SNP annotation).

a)

| Trait | PC1 | | | IQ-adjusted PC1 | | |
| --- | --- | --- | --- | --- | --- | --- |
| Dataset | P-value | Weighted Z score | Beta^a^ | P-value | Weighted Z score | Beta^a^ |
| CLDRC-RD | 9.11 x 10^-3^ | -1.41 | -0.311 | 5.9 x 10^-3^ | -1.5 | -0.315 |
| UK-RD | 1.21 x 10^-6^ | -3.4 | -0.435 | 3.67 x 10^-7^ | -3.53 | -0.436 |
| SLIC | 0.707 | -0.14 | -0.072 | 0.877 | 0.06 | 0.027 |
| CLDRC-ADHD | 0.655 | -0.13 | -0.119 | 0.606 | -0.15 | -0.13 |
| Meta-Analysis | 3.86 x 10^-7^ | -5.08 | NA^b^ | 3.01 x 10^-7^ | -5.12 | NA^b^ |

b)

| Trait | PC1 | | | IQ-adjusted PC1 | | |
| --- | --- | --- | --- | --- | --- | --- |
| Dataset | P-value | Weighted Z score | Beta^a^ | P-value | Weighted Z score | Beta^a^ |
| CLDRC-RD | 5.61 x 10^-3^ | -1.5 | -0.307 | 0.021 | -1.26 | -0.234 |
| UK-RD | 2.78 x 10^-4^ | -2.54 | -0.348 | 1.16 x 10^-3^ | -2.25 | -0.305 |
| SLIC | 0.072 | -0.66 | -0.34 | 0.232 | -0.44 | -0.197 |
| CLDRC-ADHD | 0.26 | -0.33 | -0.204 | 0.195 | -0.38 | -0.233 |
| Meta-Analysis | 5.01 x 10^-7^ | -5.03 | NA^b^ | 1.5 x 10^-5^ | -4.33 | NA^b^ |

**Table S1.** Contribution of each GWAS to the strength of the association in the PC1 and IQ-adjusted PC1 meta-analysis, for the top association signals **a)** rs59197085 (7q32.1) and **b)** rs5995177 (22q12.3). These are represented by PLINK univariate QFAM p-values and beta regression coefficients for each GWAS, and by corresponding weighted Z-scores, as computed by METAL sample size based algorithm (Willer et al. 2010). The sign of z scores and beta values refer to the allelic trend of the minor allele (A in both cases). ^a^ Although beta values computed by QFAM are not adjusted for family structure, they are reported in the table as a term of comparison for effect sizes. ^b^ Not Applicable, since the METAL sample size based algorithm computes a global weighted Z score (but not a Beta coefficient).

**References**

Johnson, A.D., Handsaker, R.E., Pulit, S.L., Nizzari, M.M., O'Donnell, C.J. & de Bakker, P.I.W. (2008) SNAP: a web-based tool for identification and annotation of proxy SNPs using HapMap. *Bioinformatics,* **24,** 2938-2939.

R Core Team (2013) R: A Language and Environment for Statistical Computing. R Foundation for Statistical Computing, Vienna, Austria.

Willer, C.J., Li, Y. & Abecasis, G.R. (2010) METAL: fast and efficient meta-analysis of genomewide association scans. *Bioinformatics,* **26,** 2190-2191.
