# Supplementary material for: Genome-wide screening for DNA variants associated with reading and language traits
Source: Genes Brain Behav. 2014 Aug 29;13(7):686–701. doi: 10.1111/gbb.12158 (PMC4165772; doi:10.1111/gbb.12158)
Supplement: Supplementary file 3 — Appendix S3: Analyses based on PC1read and IQ-adjusted PC1read (trait loadings on PC1read, Manhattan plots, QQ plots and top associations) [file gbb0013-0686-sd3.docx]

***Supplementary Material S3***

**Table S3a.** Phenotypic measures used for PC1_read_ computation within each dataset and their relative loadings on PC1_read_. The available sample sizes are also indicated (N).

^a^ Legend: WRead = word reading; WSpell = word spelling

| Trait^a^ | CLDRC-RD | UK-RD | SLIC | CLDRC-ADHD |
| --- | --- | --- | --- | --- |
| WRead | 0.938 | 0.932 | 0.967 | 0.895 |
| WSpell | 0.938 | 0.932 | 0.967 | 0.895 |
| PC1_read_ (N) | 558 | 925 | 271 | 159 |
| IQ-adjusted PC1_read_ (N) | 558 | 888 | 270 | 159 |

**Table S3 b, c.** Top association signals (p < 1x10^-5^) in the **b)** PC1_read_ and **c)** IQ-adjusted PC1_read_ genome-wide association scan meta-analysis.  ^a^ The direction of effect of Allele1 is reported for datasets in the following order: CLDRC-RD, UK-RD, SLIC, CLDRC-ADHD. ^b^ Physical distance (kb) from closest genes (in a ±10kb range from each marker) is indicated, along with orientation based on the direction of transcription ("-" = upstream of 5'-UTR, "+" = downstream of 3'-UTR).

b)

| Chr | SNP | Position (hg19) | Allele1 | Allele2 | Freq Allele1 (%) | P-value | Direction^a^ | Gene (distance)^b^ | Variant type |
| --- | --- | --- | --- | --- | --- | --- | --- | --- | --- |
| 6 | rs56139919 | 155914380 | a | g | 1.806 | 2.02 x 10^-6^ | +++- | no gene |  |
| 22 | rs5995177 | 36309553 | a | g | 8.049 | 3.56 x 10^-6^ | ---- | RBFOX2(0) | intronic |
| 12 | rs10774547 | 120862716 | t | c | 67.02 | 4.76 x 10^-6^ | ++-+ | no gene |  |
| 11 | rs118151645 | 133866086 | c | g | 96.437 | 5.57 x 10^-6^ | ++++ | no gene |  |
| 11 | rs2275998 | 66326581 | t | c | 80.97 | 5.89 x 10^-6^ | ---- | CTSF(+4.353kb)\|ACTN3(0) | intronic |
| 11 | rs2229455 | 66328055 | a | g | 80.97 | 6.64 x 10^-6^ | ---- | CTSF(+2.879kb)\|ACTN3(0) | exonic, synonymous |
| 12 | rs4766962 | 120863235 | a | t | 66.07 | 6.99 x 10^-6^ | ++-+ | no gene |  |
| 6 | rs7765720 | 155830568 | c | g | 98.102 | 7.03 x 10^-6^ | ---+ | no gene |  |
| 12 | rs7970534 | 120862195 | c | g | 33.94 | 7.33 x 10^-6^ | --+- | no gene |  |
| 6 | rs113262260 | 155854928 | a | g | 1.899 | 8.16 x 10^-6^ | +++- | no gene |  |
| 7 | rs3800560 | 128461094 | t | c | 7.971 | 8.46 x 10^-6^ | ---- | FLNC(-9.388)\|CCDC136(0) | intronic |
| 7 | rs58845495 | 128462847 | t | c | 92.029 | 9.02 x 10^-6^ | ++++ | FLNC(-7.635)\|CCDC136(+0.664) |  |
| 11 | rs2229456 | 66328741 | a | c | 80.97 | 9.32 x 10^-6^ | ---- | CTSF(+2.193kb)\|ACTN3(0) | exonic, missense |
| 7 | rs59197085 | 128460756 | a | g | 7.971 | 9.78 x 10^-6^ | ---- | FLNC(-9.726)\|CCDC136(0) | intronic |
| 12 | rs4767891 | 120863422 | a | g | 33.93 | 9.91 x 10^-6^ | --+- | no gene |  |

c)

| Chr | SNP | Position (hg19) | Allele1 | Allele2 | Freq Allele1 (%) | P-value | Direction^a^ | Gene (distance)^b^ | Variant type |
| --- | --- | --- | --- | --- | --- | --- | --- | --- | --- |
| 11 | rs1496243 | 133620944 | a | g | 33.34 | 3.41 x 10^-7^ | ---- | no gene |  |
| 11 | rs4937830 | 133645903 | c | g | 64.82 | 5.53 x 10^-7^ | ++++ | no gene |  |
| 11 | rs10894745 | 133647016 | a | g | 34.77 | 1.21 x 10^-6^ | ---- | no gene |  |
| 11 | rs7944602 | 133612417 | a | g | 33.48 | 1.32 x 10^-6^ | ---- | no gene |  |
| 11 | rs4936208 | 133644469 | t | c | 34.77 | 1.38 x 10^-6^ | ---- | no gene |  |
| 11 | rs4937829 | 133642497 | a | g | 34.73 | 1.56 x 10^-6^ | ---- | no gene |  |
| 11 | rs2220960 | 133639946 | a | g | 65.3 | 1.87 x 10^-6^ | ++++ | no gene |  |
| 6 | rs56139919 | 155914380 | a | g | 1.806 | 2.23 x 10^-6^ | +++- | no gene |  |
| 12 | rs10774547 | 120862716 | t | c | 67.02 | 3.39 x 10^-6^ | ++-+ | no gene |  |
| 11 | rs4936207 | 133631687 | a | c | 35.66 | 3.45 x 10^-6^ | ---- | no gene |  |
| 12 | rs4766962 | 120863235 | a | t | 66.07 | 3.60 x 10^-6^ | ++++ | no gene |  |
| 11 | rs10431101 | 133615843 | t | c | 65.25 | 3.65 x 10^-6^ | ++++ | no gene |  |
| 11 | rs6590728 | 133618314 | t | c | 34.52 | 3.79 x 10^-6^ | ---- | no gene |  |
| 12 | rs7970534 | 120862195 | c | g | 33.94 | 4.03 x 10^-6^ | ---- | no gene |  |
| 12 | rs4767891 | 120863422 | a | g | 33.93 | 4.32 x 10^-6^ | ---- | no gene |  |
| 7 | rs58845495 | 128462847 | t | c | 92.029 | 6.54 x 10^-6^ | ++-+ | FLNC(-7.635)\|CCDC136(+0.664) |  |
| 6 | rs16890716 | 80131140 | a | g | 84.71 | 6.57 x 10^-6^ | ++++ | no gene |  |
| 6 | rs17800074 | 80126873 | t | c | 15.21 | 7.26 x 10^-6^ | ---- | no gene |  |
| 6 | rs62411317 | 80128434 | a | c | 84.76 | 7.38 x 10^-6^ | ++++ | no gene |  |
| 7 | rs3800560 | 128461094 | t | c | 7.971 | 8.56 x 10^-6^ | --+- | FLNC(-9.388)\|CCDC136(0) | intronic |
| 6 | rs62411314 | 80120593 | a | t | 15.19 | 8.71 x 10^-6^ | ---- | no gene |  |
| 7 | rs59197085 | 128460756 | a | g | 7.971 | 9.11 x 10^-6^ | --+- | FLNC(-9.726)\|CCDC136(0) | intronic |

a)


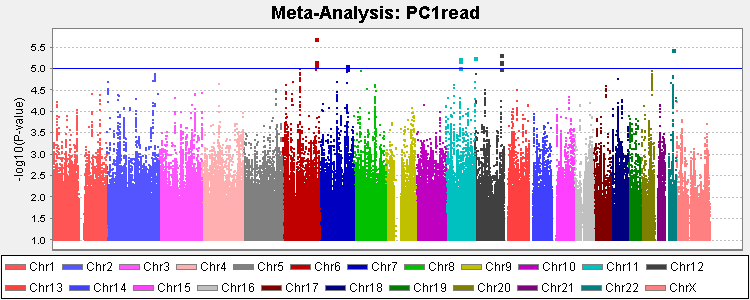


b)


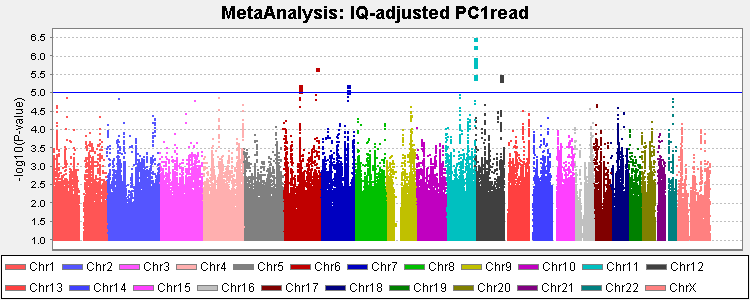


**Figure S3 a, b.** Manhattan plots of the **a)** PC1_read_ and **b)** IQ-adjusted PC1_read_ genome-wide association scan meta-analysis. The blue lines represent the nominal suggestive (p = 1x10^-5^) significance threshold.

c)


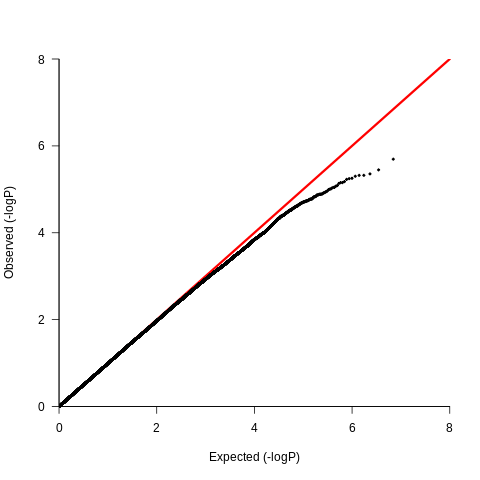


d)


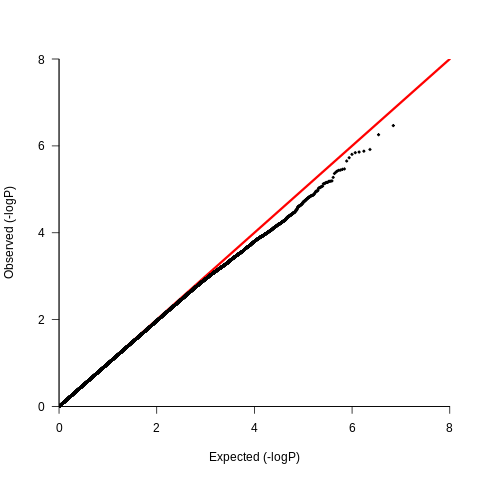


**Figure S3c, d.** QQ-plots of the **c)** PC1_read_ and **d)** IQ-adjusted PC1_read_ genome-wide association scan meta-analysis. The plots were drawn through a dedicated R script (R core Team 2013, <http://www.r-project.org/>).

**References**

R Core Team (2013) R: A Language and Environment for Statistical Computing. R Foundation for Statistical Computing, Vienna, Austria.
