# Supplementary material for: Genome-wide screening for DNA variants associated with reading and language traits
Source: Genes Brain Behav. 2014 Aug 29;13(7):686–701. doi: 10.1111/gbb.12158 (PMC4165772; doi:10.1111/gbb.12158)
Supplement: Supplementary file 4 — Appendix S4: Genotype calls and QC protocols; correlation patterns of reading and language measures; statistical analyses, commands and parameters. [file gbb0013-0686-sd4.docx]

***Supplementary Methods***

**Genotype Calls and QC.**

In all the datasets both blood and saliva (including Oragene® kit) samples were collected, and within datasets some samples were genotyped from blood DNA while others were genotyped from saliva DNA. Comparable call rates and concordance rates between blood and saliva samples have been reported in the literature (Abraham et al. 2012).

*Genotype Calls*

*UK-RD*

For 200 subjects, referred to as *UK-RD_small* hereafter: Genotype calls were generated using Illumina® BeadStudio software from Illumina® HumanHap 550k v1 chip. Default settings as described in other studies (Scerri et al., 2011a) were used. 550,927 SNPs were finally called.

For an additional 818 samples, called *UK-RD_big* hereafter: Genotyping was implemented on the Illumina® Human OmniExpress (v12, manifest H, 730k) array. Genotype calls were made through Illumina® GenomeStudio software according to the following protocol:

1. all the SNPs mapped as "Y" (Y chromosome) and "0" (not mapped) were zeroed (i.e. set to missing);
2. samples with genotyping success rate < 95% were discarded;
3. SNPs with call frequency < 100% were re-clustered (i.e. their intensity data were re-plotted, in order to get better quality of the calls);
4. SNPs with call frequency < 99% were zeroed;
5. samples with updated genotyping success rate < 98% were excluded;
6. SNPs with *Cluster Sep* (i.e. measure of the cluster separation for a SNP, that ranges between 0 and 1 and indicates how well the intensity signals of the different genotypes are distinguishable) < 0.3 were zeroed.

Each passage was followed by an update in SNP/sample statistics, in order to improve the quality of the genotype calls and of the samples. At the end of this procedure, 716,044 SNPs were finally called.

*SLIC*

Samples were genotyped using the Illumina® Human Omni-Express (v12.1, manifest C) array, within the GenomeStudio software. Samples were randomized across plates, with probands and co-siblings being spread evenly across plates. Also sample types (blood vs saliva) have been randomized across plates and we checked for systematic differences between genotype and allele frequencies both between plates and samples types and all were non-significant. 47 samples were duplicated across plates (concordance rate = 0.98968). SNPs and samples with a genotype success rate < 95% and/or heterozygosity rates ±2SD from the mean were removed, as were all SNPs with a Minor Allele Frequency (MAF) < 1%. SNPs with a *Gen Train* score (i.e. a number between 0 and 1 indicating how well the samples clustered for a specific locus) < 0.5 were removed. Since parents were also genotyped, SNPs and samples with an error rate ≥ 1%, as estimated by impossible inheritances within families, were removed (SLIC 2002; 2004; Newbury et al., 2009). A total of 630,167 SNPs were called.

*CLDRC*

Genotype calls were generated from Illumina® Human OmniExpress (v12, manifest H, 730k) array, using GenomeStudio software. The same protocol followed for *UK-RD_big* was used, finally resulting in genotypes for 683,242 total SNPs before quality control.

*Genotype quality control (QC)*

*UK-RD*

*Pre-imputation QC.* Since the two subsets of the *UK-RD* dataset had been genotyped on two different Illumina® platforms (as mentioned above), they were analyzed separately before imputation. However, in order to check for the absence of population stratification in the whole *UK-RD* dataset due to the different arrays used, the two subsets were temporarily merged and underwent a Multi-Dimensional Scaling (MDS) analysis of genome-wide SNP data (extracting the first 20 dimensions) on a subset of unrelated individuals (one subject per family selected from the whole dataset). This analysis revealed no effects of the factor mentioned above and received further support by the high genotype concordance rate (99.98%) of 34 duplicate samples genotyped in both subsets.

In *UK-RD_small*, 3 samples with low call rate (<98%) and 1 genome-wide homozygosity outlier (i.e. showing an extremely low homozygosity, which may suggest a bad quality of the DNA sample) were excluded. No Identity By Descent (IBD) sharing or sex inconsistencies between reported and genetically inferred information, nor MDS outliers (Figure S4a), were detected within this subset. We filtered out all the SNPs deviating from Hardy-Weinberg Equilibrium (HWE, p-val < 1x10^-6^, 12,631 SNPs) and with MAF < 1% (23,467 SNPs) in the whole subset (all unrelated individuals). 82,052 variants with call frequency < 99% were discarded.

In *UK-RD_big*, 7 samples with IBD sharing inconsistencies (half-siblings, unrelated samples showing cryptic relatedness or MZ twins); 8 sex mismatches (including 3 X chromosome abnormalities carriers) and 3 homozygosity outliers were excluded, along with 2 outliers in the MDS analysis on a subset of unrelated individuals (one subject per family, Figure S4b). All the samples had a call rate ≥ 98%. All the SNPs deviating from HWE (p-val < 1x10^-6^, 191 SNPs) and with MAF < 1% (77,342 SNPs) as calculated within the subset of unrelated individuals (one subject per family) were filtered out. No variants had call frequency < 99%.

A further IBD sharing check on the whole dataset revealed an inconsistency on one of the duplicated samples that had already been excluded in *UK-RD_big* but not in *UK-RD_small*, from which it was discarded.

*Post-imputation QC*. To ensure a high quality of imputation, imputed SNPs with r^2^ (squared correlation between the allele count estimated for a given SNP by the imputation algorithm and the allele count that would be expected if the genotype of that SNP was observed without error) < 0.3 were discarded, and all the individual genotypes with quality score (estimated probability that an imputed genotype will match an experimental genotype) < 0.9 were set to missing. Then the two subsets were merged into the definitive *UK-RD* dataset (N = 959): 2,779 SNPs failed the HWE test (p-val < 5x10^-6^) and 1,980,500 had a MAF < 1%, in a subset of unrelated individuals (one subject per family); 1,704,412 SNPs were finally excluded due to call frequency < 95%, resulting in a final total of 6,190,549 SNPs analyzed in *UK-RD*. All the samples had a call rate ≥ 95%. MDS and IBD sharing analyses of imputed data confirmed the consistency with genotyped data (concordance rate before vs after imputation 99.96%, and 99.89% between the duplicated samples genotyped and imputed separately in the two subsets).

*SLIC*

*Pre-imputation QC.* Out of 548 subjects, 9 were excluded for sex chromosome abnormalities and 1 for X chromosome call rate <95% (Newbury, personal communication); 9 for genomic call-rate < 98%; 17 for IBD sharing typical of half-siblings (19-31%) when they had been reported as full siblings; 3 sex mismatches and 2 outliers on genome-wide homozygosity. An MDS analysis of genome-wide SNP data was run on a subset of unrelated individuals (one subject per family): 2 outliers were detected and excluded (Figure S4c), along with their 3 siblings (5 in total). We filtered out SNPs deviating from HWE (p-val < 1x10^-6^, 54 SNPs) and with MAF < 1% (1,718 SNPs) as calculated in the same subset of unrelated individuals (one subject per family), as well as (72,043) variants with call frequency < 99%.

*Post-imputation QC*. Imputed SNPs with r^2^ < 0.3 were filtered out, and all the genotypes with quality score < 0.9 were set to missing. 2,096 SNPs deviated from HWE (p-val < 5x10^-6^) and 3,260,639 had a MAF < 1% in a subset of unrelated individuals (one individual per family); 1,766,376 SNPs were excluded for call frequency < 95%, leading to a final total of 6,240,842 SNPs analyzed. All the samples showed a call rate ≥ 95%. MDS and IBD sharing analyses of imputed data confirmed their consistency with directly genotyped data, as did the concordance rate between imputed and genotyped data (99.97%).

*CLDRC*

*Pre-imputation QC*. Since *CLDRC-RD* and *CLDRC-ADHD* belonged to the same dataset, we decided to treat them as a unique dataset in the genotype QC. Out of 749 initial *CLDRC* subjects, 11 samples with IBD sharing inconsistencies (half-siblings or unrelated samples showing cryptic relatedness), 3 sex mismatches, and 6 homozygosity outliers were discarded (all the samples had call rate ≥ 98% and there were no outliers in the MDS analysis on a subset of unrelated individuals, including one subject per family, Figure S4d). We filtered out all the SNPs deviating from HWE (p-val < 1x10^-6^, 57 SNPs) and with MAF < 1% (74,770 SNPs) in a subset of unrelated individuals (one subject per family). No variants had call frequency < 99%.

*Post-imputation QC*. Imputed SNPs with r^2^ < 0.3 were filtered out, and all the genotypes with quality score < 0.9 were set to missing. 2,166 SNPs did not pass the HWE test (p-val < 5x10^-6^) and 3,640,742 had a MAF < 1%, in a subset of unrelated individuals (one subject per family); 1,729,493 SNPs were finally excluded for call frequency < 95%. All the samples showed a call rate ≥ 95%. A total of 6,427,200 SNPs were examined in both the *CLDRC* datasets. MDS and IBD sharing analyses of imputed data confirmed the consistency with genotyped data (concordance rate 99.96% between genotyped and imputed data).

a)


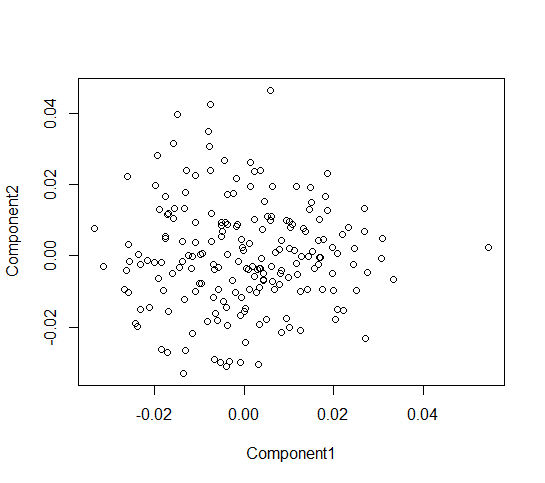


b)


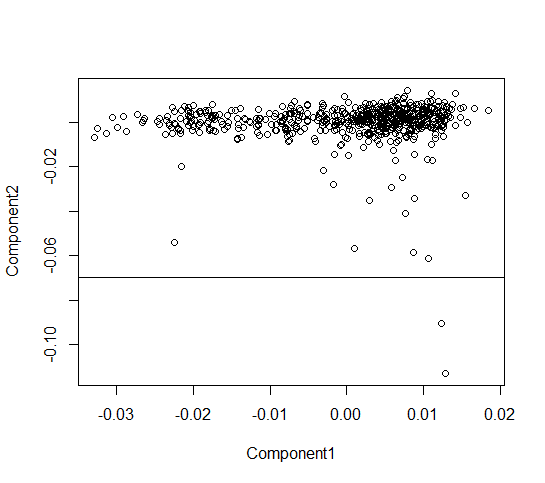


c)


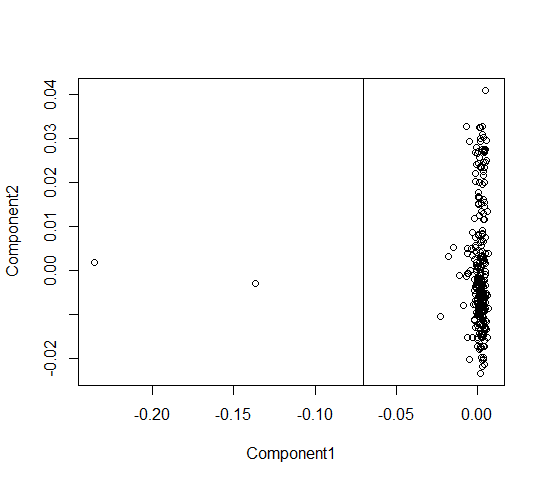


d)


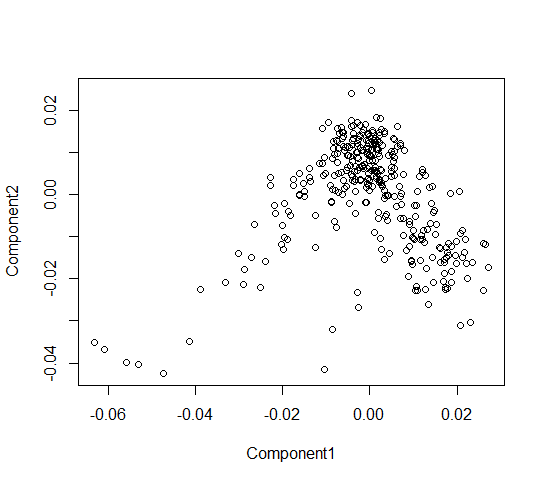


**Figure S4.** MDS analysis of **a)** *UK-RD_small* subset, **b)** *UK-RD_big* subset, **c)** SLIC and **d)** CLDRC samples on non imputed data. The most evident outliers (i.e. showing at least one of the first 3 MDS components scores extracted out of the interval [-0.7; 0.7]), were excluded (along with their co-siblings, where present). For simplicity, only the first two MDS components are shown (Component 3 did not show any outlier in any of the datasets). Imputed samples passing QC in each dataset underwent a second MDS analysis which did not reveal any outlier (data not shown).

**Correlation patterns of reading and language measures.**

**a)**

| Trait^a^ | WRead | WSpell | PD | PA | OC | PC1 | PC1_read_ |
| --- | --- | --- | --- | --- | --- | --- | --- |
| WRead | 1 | 0.749 | 0.634 | 0.552 | 0.77 | 0.92 | 0.936 |
| WSpell | 0.749 | 1 | 0.53 | 0.537 | 0.662 | 0.85 | 0.934 |
| PD | 0.634 | 0.53 | 1 | 0.656 | 0.595 | 0.794 | 0.624 |
| PA | 0.552 | 0.537 | 0.656 | 1 | 0.625 | 0.68 | 0.59 |
| OC | 0.77 | 0.662 | 0.595 | 0.625 | 1 | 0.883 | 0.767 |
| PC1 | 0.92 | 0.85 | 0.794 | 0.68 | 0.883 | 1 | 0.947 |
| PC1_read_ | 0.936 | 0.934 | 0.624 | 0.59 | 0.767 | 0.947 | 1 |

**b)**

| Trait^a^ | WRead | WSpell | NWR | ELS | RLS | PC1 | PC1_read_ |
| --- | --- | --- | --- | --- | --- | --- | --- |
| WRead | 1 | 0.869 | 0.463 | 0.679 | 0.647 | 0.903 | 0.968 |
| WSpell | 0.869 | 1 | 0.412 | 0.618 | 0.584 | 0.858 | 0.966 |
| NWR | 0.463 | 0.412 | 1 | 0.536 | 0.433 | 0.645 | 0.453 |
| ELS | 0.679 | 0.618 | 0.536 | 1 | 0.769 | 0.857 | 0.673 |
| RLS | 0.647 | 0.584 | 0.433 | 0.769 | 1 | 0.822 | 0.637 |
| PC1 | 0.903 | 0.858 | 0.645 | 0.857 | 0.822 | 1 | 0.914 |
| PC1_read_ | 0.968 | 0.966 | 0.453 | 0.673 | 0.637 | 0.914 | 1 |

**c)**

| Trait^a^ | WRead | WSpell | PD  (NWRead) | PD  (PC) | PA | OC | NWR | PC1 | PC1_read_ |
| --- | --- | --- | --- | --- | --- | --- | --- | --- | --- |
| WRead | 1 | 0.768 | 0.851 | 0.736 | 0.652 | 0.661 | 0.396 | 0.922 | 0.941 |
| WSpell | 0.768 | 1 | 0.671 | 0.583 | 0.503 | 0.691 | 0.286 | 0.819 | 0.94 |
| PD  (NWRead) | 0.851 | 0.671 | 1 | 0.759 | 0.694 | 0.596 | 0.328 | 0.896 | 0.811 |
| PD  (PC) | 0.736 | 0.583 | 0.759 | 1 | 0.73 | 0.606 | 0.336 | 0.863 | 0.703 |
| PA | 0.652 | 0.503 | 0.694 | 0.73 | 1 | 0.445 | 0.422 | 0.796 | 0.615 |
| OC | 0.661 | 0.691 | 0.596 | 0.606 | 0.445 | 1 | 0.219 | 0.768 | 0.721 |
| NWR | 0.396 | 0.286 | 0.328 | 0.336 | 0.422 | 0.219 | 1 | 0.479 | 0.36 |
| PC1 | 0.922 | 0.819 | 0.896 | 0.863 | 0.796 | 0.768 | 0.479 | 1 | 0.925 |
| PC1_read_ | 0.941 | 0.94 | 0.811 | 0.703 | 0.615 | 0.721 | 0.36 | 0.925 | 1 |

**d)**

| Trait^a^ | WRead | WSpell | PD (NWRead) | PD  (PC) | PA | OC | NWR | PC1 | PC1_read_ |
| --- | --- | --- | --- | --- | --- | --- | --- | --- | --- |
| WRead | 1 | 0.622 | 0.779 | 0.542 | 0.566 | 0.454 | 0.279 | 0.878 | 0.901 |
| WSpell | 0.622 | 1 | 0.491 | 0.405 | 0.492 | 0.572 | 0.157 | 0.769 | 0.901 |
| PD  (NWRead) | 0.779 | 0.491 | 1 | 0.556 | 0.629 | 0.373 | 0.171 | 0.829 | 0.712 |
| PD  (PC) | 0.542 | 0.405 | 0.556 | 1 | 0.499 | 0.419 | 0.091 | 0.724 | 0.534 |
| PA | 0.566 | 0.492 | 0.629 | 0.499 | 1 | 0.291 | 0.219 | 0.758 | 0.597 |
| OC | 0.454 | 0.572 | 0.373 | 0.419 | 0.291 | 1 | 0.089 | 0.637 | 0.567 |
| NWR | 0.279 | 0.157 | 0.171 | 0.091 | 0.219 | 0.089 | 1 | 0.318 | 0.254 |
| PC1 | 0.878 | 0.769 | 0.829 | 0.724 | 0.758 | 0.637 | 0.318 | 1 | 0.917 |
| PC1_read_ | 0.901 | 0.901 | 0.712 | 0.534 | 0.597 | 0.567 | 0.254 | 0.917 | 1 |

**Table S4.** Pairwise trait correlation of reading and language measures in **a)** UK-RD, **b)** SLIC, **c)** CLDRC-RD, and **d)** CLDRC-ADHD datasets. These were computed separately within each dataset, as the median Pearson’s correlation over 100 repeat random samplings of one individual from each independent sibship. For each dataset, also pairwise correlations for PC1 and PC1_read_ were tested.

^a^ Legend: WRead = word reading; WSpell = word spelling; PD = phonological decoding (NWRead = nonword reading and PC = phonological choice); PA = phoneme awareness; OC = orthographic coding; NWR = nonword repetition; ELS/RLS = expressive/receptive language score; PC1 = first principal component derived from all the reading and language measures available in each dataset (except for PA in UK-RD, which was excluded from the PCA due to the low number of measures available); PC1_read_ = first principal component derived from word reading and spelling only. For more information on the measures and a description of the skills assessed, see Tables 2–5 in the manuscript.

**Statistical analyses: commands and parameters.**

*PLINK QFAM (family-based association tests for quantitative traits)* *analysis*

Sibling-based genome-wide association analysis of PC1 scores was conducted using PLINK v1.07 (Purcell et al., 2007) *--qfam-total* analysis, a permutation-based method correcting for subject relatedness. In this analysis the association between a quantitative trait and a SNP is tested by regressing the trait score on the SNP genotype in an additive model (as in the *--linear* analysis). However, to adjust for sample relatedness, a high number of permutations (i.e. label-swapping of phenotypes/genotypes) are run and after each permutation a linear regression associated p-value is produced. All the p-values are then plotted and an empirical permuted p-value for each SNP is computed (defined as the probability to obtain a statistic lower than or equal to the one obtained in the first "no permutation" test). The *--qfam-total* procedure is based on the between/within model reported by Fulker et al. (1999) and Abecasis et al. (2000): each genotype score is decomposed in a within family and between-family component, which undergo permutations in the same family *(--qfam-within*) and between different families *(--qfam-between*), respectively. Then the two components are summed to create a new total genotype score, which is tested for association (further details can be found in the PLINK tutorial). An adaptive permutation procedure (*--aperm*) was used, with the following parameters:

- Minimum number of permutations per SNP *1,000*
- Maximum number of permutations per SNP *1,000,000,000*
- Alpha (determining the threshold for pruning p-values) *0*
- Beta (determining the width of confidence interval on empirical p-value) *0.01*
- Initial interval (nr of permutations) to prune SNP test list *100*
- Rate of increase of the initial interval to prune SNP test list *0.001*

Detailed explanations of these arguments can be found in the PLINK tutorial (<http://pngu.mgh.harvard.edu/~purcell/plink/>).

*SNP-based Meta-analysis (METAL)*

A sample size-based meta-analysis was run in METAL (Willer et al., 2010). This method consists of computing an overall z-score for each SNP as a weighted sum of z-scores, determined by the p-value, the direction of the effect and the sample size of each study involved in the meta-analysis.

To this purpose, the default *SCHEME SAMPLESIZE* command was used, along with the *ANALYZE HETEROGENEITY* option to check for the homogeneity of effect sizes across the different datasets (see <http://www.sph.umich.edu/csg/abecasis/Metal/index.html> for further details).

*Gene-based Meta-analysis (VEGAS)*

VEGAS performs gene-based association tests for all the 17,787 autosomal genes present in the UCSC Genome Browser map (hg18 assembly), assigning SNPs to genes and combining their effects taking into account the linkage disequilibrium (LD) structure of the genes (Liu et al. 2010). A Bonferroni-corrected significance threshold was set at p <2.8×10^−6^ to account for the number of genes tested. Gene boundaries were extended up to 50 kb upstream/downstream of 5'-/3'-UTRs, respectively (to include possible variants located in regulatory regions), while the LD patterns for each gene were inferred from the SNP data of the HapMap CEU population (release R2; The International HapMap 3 Consortium, 2010; http://www.hapmap.org/). These options were implemented in the following (default) commands:

*-lower 50000* (5'-UTR extension in bp)

*-upper 50000* (3'-UTR extension in bp)

*-pop HapMap CEU* (population of reference for LD inference)

A detailed explanation of the commands is available at <http://gump.qimr.edu.au/VEGAS/>.

*Pathway-based Meta-analysis (INRICH)*

The INRICH tool (Lee et al., 2012) for pathway-based association tests takes a set of independent associated genomic intervals and tests them for the enrichment of predefined gene sets (i.e. pathways) through a permutation-based approach. This required extrapolating the associated genomic intervals from the meta-analysis results file through the PLINK *--clump* command, using the following arguments:

*--clump-p1 0.001* (p-value threshold for index SNPs = 0.001)

*--clump-p2 0.01* (p-value threshold for clumped SNPs = 0.01)

*--clump-r2 0.5* (LD (r-squared) threshold for clumping = 0.5)

*--clump-kb 250* (Physical (kb) threshold for clumping = 250)

Detailed explanations of these arguments can be found in the PLINK tutorial (<http://pngu.mgh.harvard.edu/~purcell/plink/>).

Three composite candidate pathways -axon guidance, neuronal migration and steroid sex hormone biology- were tested, using the following INRICH options:

*-w 50000 (gene boundaries extension, bp)*

*-i 10 (minimum nr of genes in tested pathways)*

*-j 400 (maximum nr of genes in tested pathways)*

*-p 1 (list gene sets with empirical p-value ≤ 1)*

*-z 3 (consider only gene sets with ≥ 3 overlapping intervals)*

The INTERVALS test (default analysis examining enriched association signals for pre-defined sets of genetic variants) and Entrez hg18 gene map for the reference gene file (with genomic coordinates updated to hg19 through the UCSC LiftOver Tool, <http://genome.ucsc.edu/cgi-bin/hgLiftOver>) were used. Also in this case we extended gene boundaries ±50 kb from the 5'- and 3'-UTRs, to include regulatory regions in the analysis (please notice that the extension is indicated in bp since a bug was found in the current release of the software; Lee, personal communication). For all the other options and commands see the INRICH user manual at <http://atgu.mgh.harvard.edu/inrich/started.html>.

*PLINK Multivariate analysis of top hits*

PLINK Multivariate (Ferreira & Purcell, 2009) is a PLINK v1.06 plugin which runs multivariate association tests with several continuous phenotypic traits. Considering a SNP and a set of continuous traits, this tool executes a Canonical Correlation Analysis (CCA), extracting the linear combination of traits that explains the maximum amount of covariation between the SNP and the traits analyzed. This produces as output a single p-value per SNP, representing the significance of multivariate association, and a set of loadings for each trait analyzed, corresponding to the correlation between the trait and the latent variable extracted from all the traits analyzed, and representing the contribution of the trait to the multivariate association. See <https://genepi.qimr.edu.au/staff/manuelF/multivariate/main.html> for detailed explanation.

To adjust for sample relatedness in the datasets, a permutation-based *--mqfam-total* analysis was run. This is a multivariate version of the *–qfam-total* test described above. An adaptive permutation procedure (*--aperm*) was used also in this case, with the same parameters settings used in the univariate QFAM total association test on PC1 scores (see *PLINK QFAM* section above).

**References**

Abecasis, G.R., Cardon, L.R. & Cookson, W.O.C. (2000) A General Test of Association for Quantitative Traits in Nuclear Families. *Am J Hum Genet ,* **66,** 279–292.

Abraham, J., Maranian, M., Spiteri, I., Russell, R., Ingle, S., Luccarini, C., Earl, H., Pharoah, P., Dunning, A. & Caldas, C. (2012) Saliva samples are a viable alternative to blood samples as a source of DNA for high throughput genotyping. *BMC Med Genomics*, **5**, 19.

Ferreira, M.A. & Purcell, S.M. (2009) A multivariate test of association. *Bioinformatics*, **25**, 132-133.

Fulker, D.W., Cherny, S.S., Sham, P.C. & Hewitt, J.K. (1999) Combined Linkage and Association Sib-Pair Analysis for Quantitative Traits. *Am J Hum Genet ,* **64,** 259-267.

Lee, P.H., O'Dushlaine, C., Thomas, B. & Purcell, S.M. (2012) INRICH: interval-based enrichment analysis for genome-wide association studies. *Bioinformatics,* **28,** 1797-1799.

Liu, J.Z., McRae, A.F., Nyholt, D.R., Medland, S.E., Wray, N.R., Brown, K.M., Hayward, N.K., Montgomery, G.W., Visscher, P.M., Martin, N.G. & Macgregor, S. (2010) A versatile gene-based test for genome-wide association studies. *Am J Hum Genet,* **87,** 139-145.

Newbury, D.F., Winchester, L., Addis, L., Paracchini, S., Buckingham, L.L., Clark, A., Cohen, W., Cowie, H., Dworzynski, K., Everitt, A., Goodyer, I.M., Hennessy, E., Kindley, A.D., Miller, L.L., Nasir, J., O'Hare, A., Shaw, D., Simkin, Z., Simonoff, E., Slonims, V., Watson, J., Ragoussis, J., Fisher, S.E., Seckl, J.R., Helms, P.J., Bolton, P.F., Pickles, A., Conti-Ramsden, G., Baird, G., Bishop, D.V. & Monaco, A.P. (2009) CMIP and ATP2C2 modulate phonological short-term memory in language impairment. *Am J Hum Genet,* **85,** 264-272.

Purcell, S., Neale, B., Todd-Brown, K., Thomas, L., Ferreira, M.A., Bender, D., Maller, J., Sklar, P., de Bakker, P.I., Daly, M.J. & Sham, P.C. (2007) PLINK: a tool set for whole-genome association and population-based linkage analyses. *Am J Hum Genet,* **81,** 559-575.

The SLI Consortium (2002) A Genomewide Scan Identifies Two Novel Loci Involved in Specific Language Impairment* *Members of the consortium are listed in the Appendix. *Am J Hum Genet ,* **70,** 384-398.

The SLI Consortium (2004) Highly significant linkage to the SLI1 locus in an expanded sample of individuals affected by specific language impairment. *Am J Hum Genet,* **74,** 1225-1238.

Scerri, T.S., Brandler, W.M., Paracchini, S., Morris, A.P., Ring, S.M., Richardson, A.J., Talcott, J.B., Stein, J. & Monaco, A.P. (2011a) PCSK6 is associated with handedness in individuals with dyslexia. *Hum Mol Genet,* **20,** 608-614.

Willer, C.J., Li, Y. & Abecasis, G.R. (2010) METAL: fast and efficient meta-analysis of genomewide association scans. *Bioinformatics,* **26,** 2190-2191.
